# Association of genetic risk, lifestyle, and their interaction with obesity and obesity-related morbidities

**DOI:** 10.1016/j.cmet.2024.06.004

**Published:** 2024-07-02

**Authors:** Min Seo Kim, Injeong Shim, Akl C. Fahed, Ron Do, Woong-Yang Park, Pradeep Natarajan, Amit V. Khera, Hong-Hee Won

**Affiliations:** 1 Department of Digital Health, Samsung Advanced Institute for Health Sciences & Technology (SAIHST), Sungkyunkwan University, Seoul 06355, Republic of Korea; 2 Cardiovascular Disease Initiative, Broad Institute of MIT and Harvard, Cambridge, MA 02141, USA; 3 Cardiovascular Research Center, Massachusetts General Hospital, Boston, MA 02114, USA; 4 Department of Medicine, Harvard Medical School, Boston, MA 02115, USA; 5 The Charles Bronfman Institute for Personalized Medicine, Icahn School of Medicine at Mount Sinai, New York, NY 10029, USA; 6 Department of Genetics and Genomic Sciences, Icahn School of Medicine at Mount Sinai, New York, NY 10029, USA; 7 Samsung Genome Institute, Samsung Medical Center, Seoul 06351, Republic of Korea; 8 Center for Genomic Medicine, Massachusetts General Hospital, Boston, MA 02114, USA; 9 Division of Cardiovascular Medicine, Department of Medicine, Brigham and Women’s Hospital, Boston, MA 02115, USA; 10 Verve Therapeutics, Boston, MA 02215, USA; 11 These authors contributed equally; 12 Lead contact

## Abstract

The extent to which modifiable lifestyle factors offset the determined genetic risk of obesity and obesity-related morbidities remains unknown. We explored how the interaction between genetic and lifestyle factors influences the risk of obesity and obesity-related morbidities. The polygenic score for body mass index was calculated to quantify inherited susceptibility to obesity in 338,645 UK Biobank European participants, and a composite lifestyle score was derived from five obesogenic factors (physical activity, diet, sedentary behavior, alcohol consumption, and sleep duration). We observed significant interaction between high genetic risk and poor lifestyles (*p*_interaction_ < 0.001). Absolute differences in obesity risk between those who adhere to healthy lifestyles and those who do not had gradually expanded with an increase in polygenic score. Despite a high genetic risk for obesity, individuals can prevent obesity-related morbidities by adhering to a healthy lifestyle and maintaining a normal body weight. Healthy lifestyles should be promoted irrespective of genetic background.

## In brief

Kim et al. demonstrate modifiable lifestyle factors can offset the determined genomic risk for obesity. Adherence to a healthy lifestyle (minimal obesogenic behaviors) is associated with a reduced risk of obesity and obesity-related morbidities across all genetic backgrounds.

## Graphical abstract

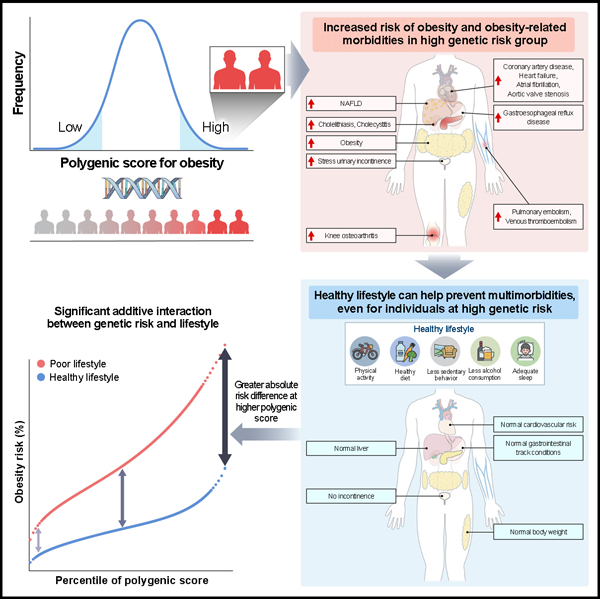

## INTRODUCTION

Obesity is a predominant non-infectious pandemic worldwide,^1^ driven by increased intake of energy-dense foods and sedentary lifestyles.^2^ The profound metabolic burden of obesity leads to various comorbidities,^3,4^ and as such, it represents a key target for global health policies and public health efforts aimed at reducing its health and economic burden.^5,6^

Obesity is largely heritable and known to be polygenic.^7,8^ Numerous metabolic pathways contribute to weight gain, and more than a thousand independent genetic variants have been associated with obesity.^7^ Against the long belief that genetic predisposition to obesity is non-modifiable,^9^ increasing evidence from gene-environment (G×E) interaction studies advocates that certain lifestyle factors, such as physical activity, may attenuate the effect of some obesity-related genes.^7,10^ However, such investigations have been limited to a few lifestyle factors and obesogenic genes such as *FTO*. It is unclear how modifiable lifestyle factors for obesity might interact with a genome-wide predisposition to obesity, measured by a polygenic score (PGS), and attenuate its burden.

Based on our previously proposed design,^11^ this study aimed to examine the extent to which modifiable lifestyle factors could offset the determined genomic risk for obesity. We used the PGS for body mass index (BMI) to quantify inherited susceptibility to obesity, as justified previously,^8^ and explored its association and interaction with lifestyle factors. As obesity is causally associated with multimorbidity, we further investigated the effect of adherence to a healthy lifestyle on the risks of 20 obesity-related morbidities (ORMs) using genetic, lifestyle, and comorbidity data from 338,645 individuals of European ancestry.

## RESULTS

### Genetic and lifestyle characteristics of study participants

**Figure 1.**
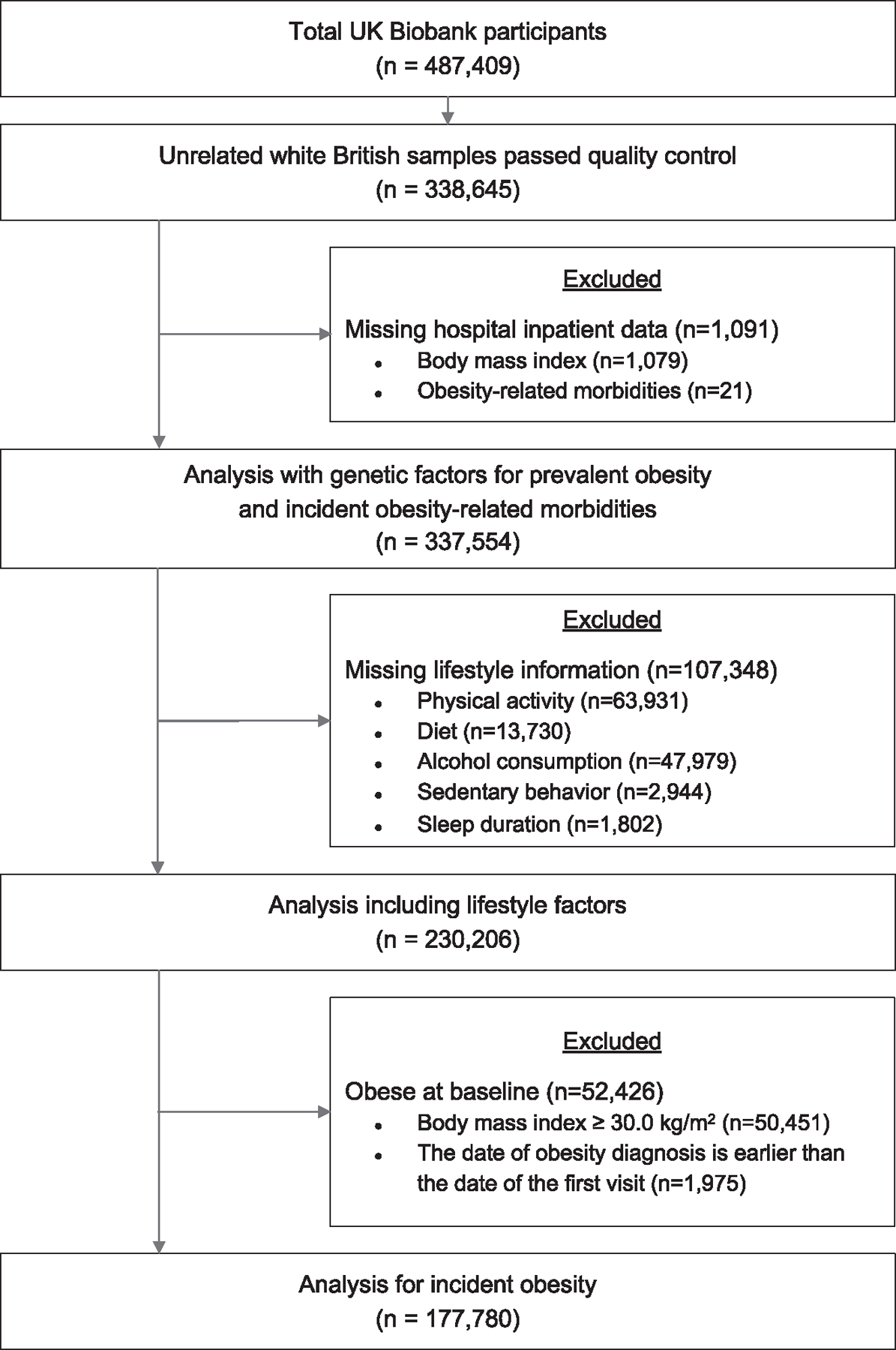
Study flow diagram A person could be excluded for more than 2 criteria.

Of 487,409 UK Biobank participants, 338,645 individuals who were unrelated, white British, and passed genetic quality control were included in the analysis. After excluding 1,079 participants without BMI information and 21 participants lacking ORM data (nine participants were excluded for missing value in BMI and ORM data), 337,554 individuals were included in the analyses (Figure 1; Table S1). Individuals with obesity exhibited higher PGS_BMI_ and fewer healthy lifestyle factors (Tables 1 and S1). The PGS_BMI_ demonstrated an association with the measured BMI and exhibited normal distribution (Figure S1). Moreover, the categories of genetic risk were proportionally distributed across lifestyle groups (Figure S1).

### Isolated effects of genetic risk and lifestyle

**Figure 2.**
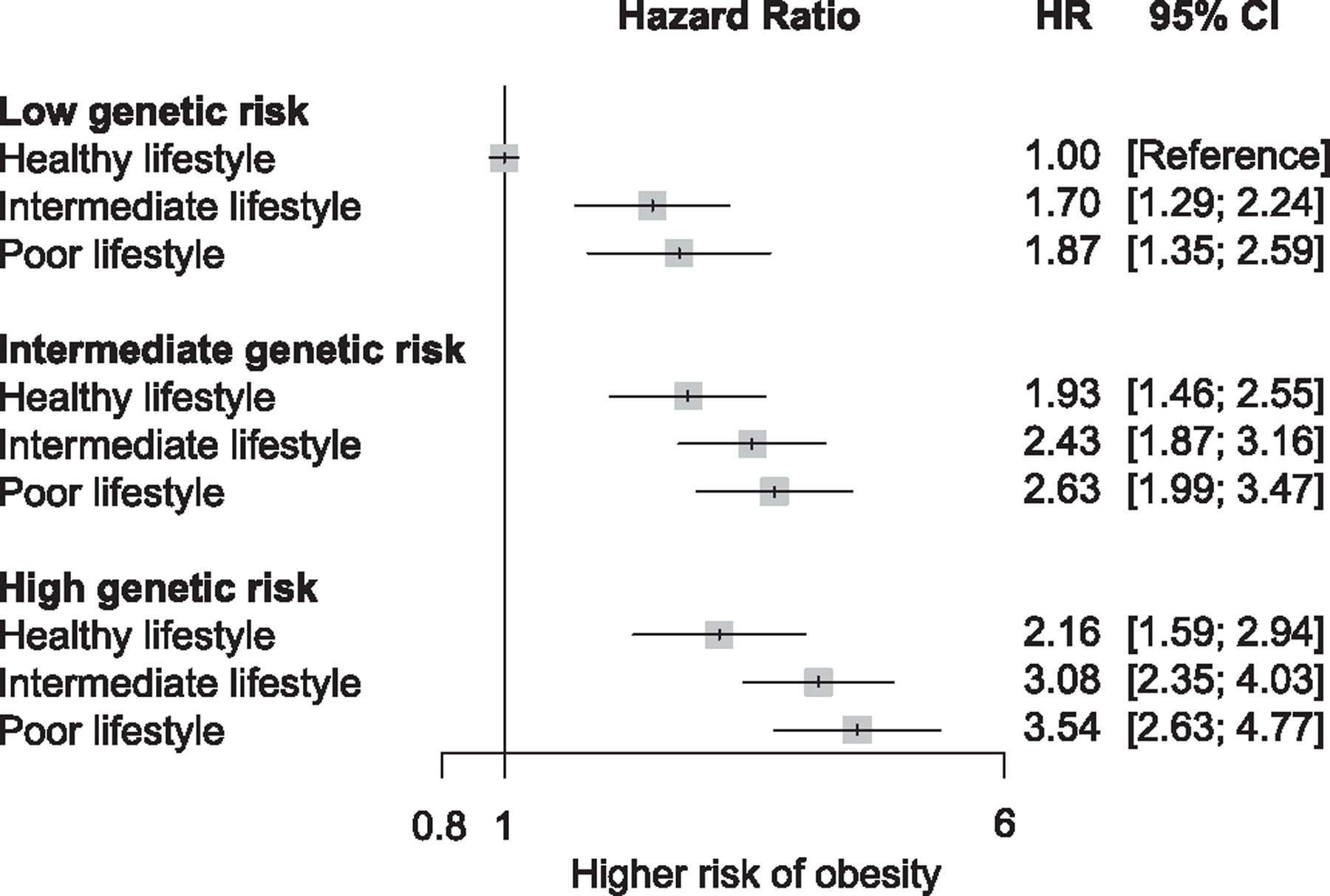
Association of genetic risk and lifestyle with incident obesity Effect estimates were derived from adjusted models. Participants with a low genetic risk and healthy lifestyle served as the reference group. HR, hazard ratio; CI, confidence interval.

High genetic risk and an unhealthy lifestyle were independently (Table 2) and jointly associated with obesity (Figure 2). The isolated effect of genetic risk on obesity (independent of lifestyle) was examined by adjusting for lifestyle groups, and high genetic risk was associated with an increased risk of incident and prevalent obesity, independent of lifestyle categories (hazard ratio [HR], 1.86; 95% confidence intervals [CI], 1.68 to 2.07; odds ratio [OR], 4.59; 95% CI, 4.43 to 4.76) (Tables 2 and S2). Similarly, the isolated effect of lifestyle on obesity (independent of genetic risk) was examined after adjusting for genetic risk groups. Poor lifestyle risk was associated with an increased risk of incident and prevalent obesity, regardless of genetic risk (HR, 1.48, 95% CI, 1.32 to 1.67; OR, 2.57; 95% CI, 2.47 to 2.67) (Tables 2 and S2). Additional factors associated with healthy lifestyle further decreased the risk of incident and prevalent obesity, even after adjusting for genetic risk and other covariates (Table S3).

### Interplay between genetic risk and lifestyle

**Figure 3.**
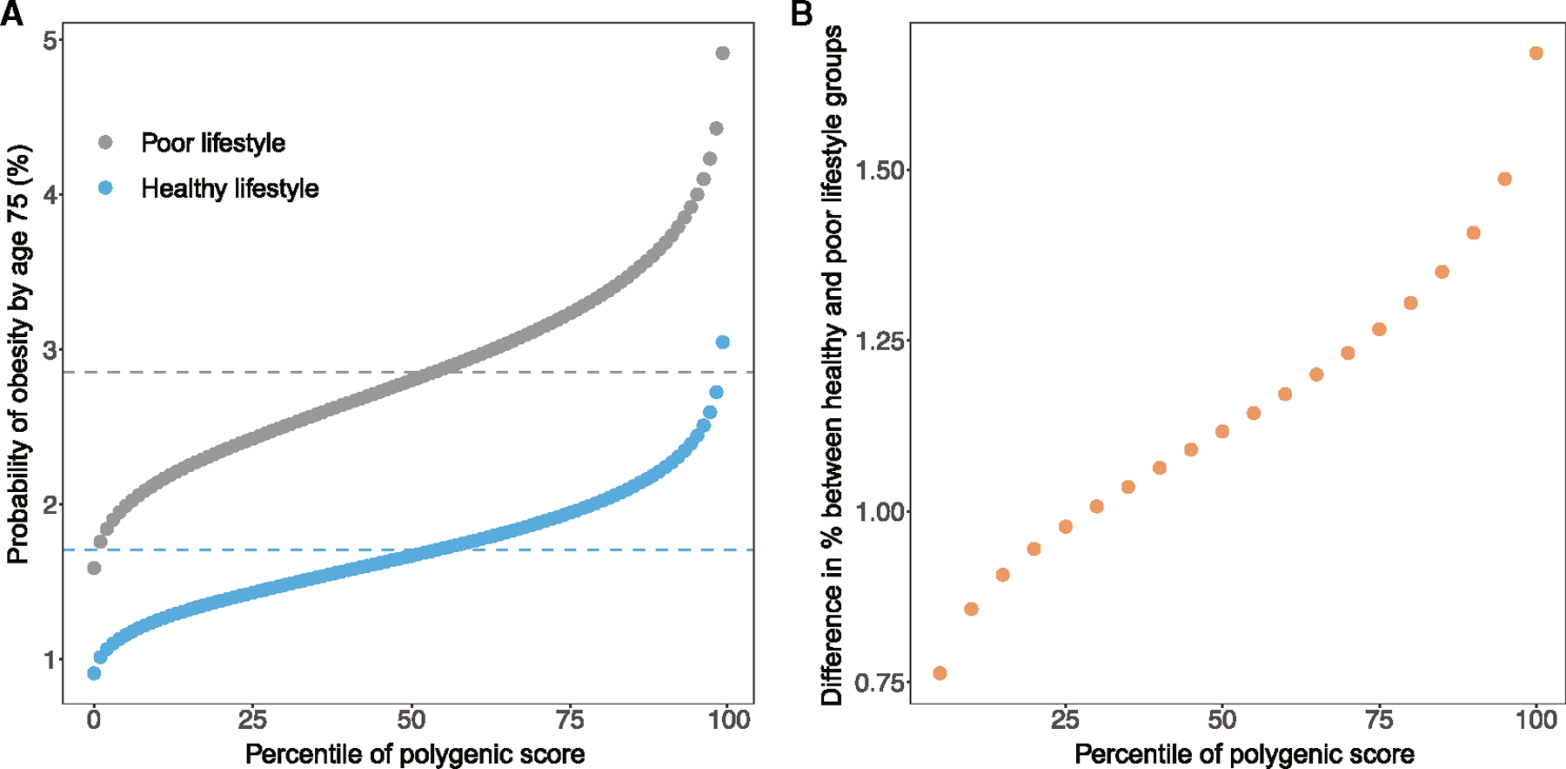
Predicted probability of obesity risk by lifestyle (A) Probability of incident obesity (%) by polygenic score for the healthy (blue) and poor (gray) lifestyle groups. (B) Differences in percentage units between the healthy and poor lifestyle groups by polygenic score.

A significant risk of obesity was observed with high genetic risk and poor lifestyle. The HR of obesity in participants with high genetic risk and poor lifestyle was 3.54 (95% CI, 2.63 to 4.77) relative to those with low genetic risk and healthy lifestyle (Figure 2). Additionally, the value decreased incrementally for each reduction in lifestyle or genetic risk category. The pattern was similar across prevalent obesity defined by BMI and other obesity indices (body fat percentage, waist circumference [WC], and waist-to-hip ratio [WHR]) as well as sex and Townsend deprivation index (TDI) subgroups (Figure S2). The median probability of obesity (%) by age 75, based on incident obesity, was 1.7% in the healthy lifestyle group and 2.8% in the poor lifestyle group (Figure 3A). The difference in the percentage unit of obesity risk (%) by age 75 between the healthy and poor lifestyle groups was 0.76 in the bottom 5th percentile of the PGS and 1.67 in the top 5th percentile of the PGS (Data S1; Figure 3B). When the predicted probability of obesity (%) by age 75 was based on prevalent obesity, the gradient was significantly substantial, and the median probability was 13.9% in the healthy lifestyle group and 30.7% in the poor lifestyle group (Figure S3). The difference in the percentage unit of obesity risk (%) by age 75 between the healthy and poor lifestyle groups was 8.5 in the bottom 5th percentile of the PGS and 22.8 in the top 5th percentile of the PGS (Data S1; Figure S3).

For the interaction analysis, we primarily used prevalent obesity to capture the full influence of genetic risk that begins from birth, similar to approaches taken in previous studies.^12,13^ The additive interaction between genetic risk and lifestyle was examined using the relative excess risk due to the interaction (RERI) (RERI > 0 indicates the presence of significant additive interactions), and the analysis revealed clear additive interactions between the two. The attributable proportions of joint effects were 56.6% (95% CI, 54.3%–59.0%) to genetic risk alone, 25.2% (23.9%–26.6%) to lifestyle alone, and 18.1% (95% CI, 17.1%–19.2%) to their interaction (Table S2). The multiplicative interaction analysis also yielded consistent results (*p*_interaction_ < 0.001) (Tables S4–S9).

When dissecting healthy lifestyles into five factors, avoiding a sedentary lifestyle was associated with the lowest likelihood of obesity, regardless of genetic risk (Table S10). In contrast, abstaining from alcohol consumption was associated the least with the odds of being obese and was heterogeneous across genetic risks. Additive interaction analysis revealed a similar pattern, in which a sedentary lifestyle was the most prominently interacting lifestyle factor with genetic risk for obesity (Table S11).

### Obesity-related morbidities

**Figure 4.**
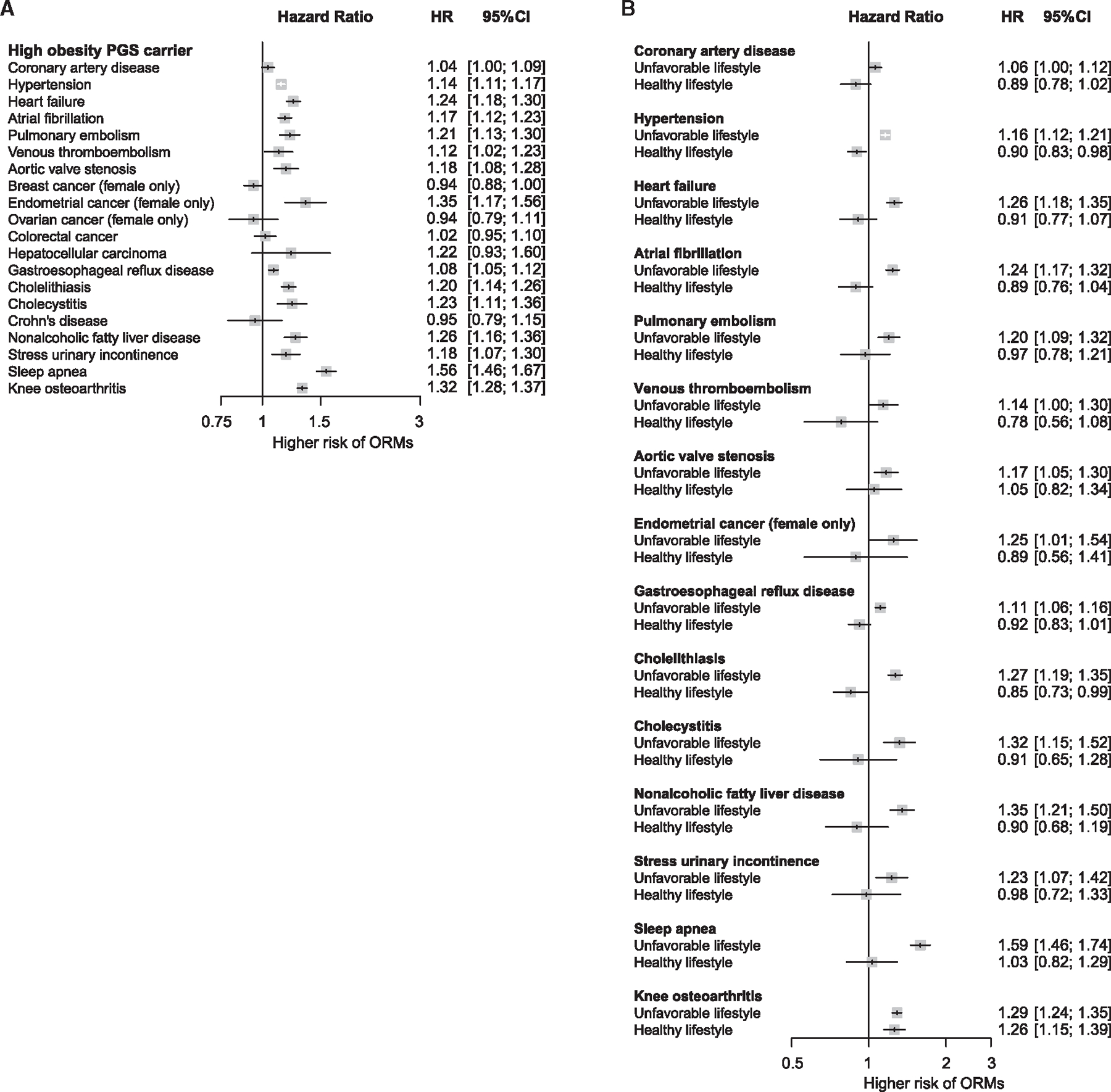
Association of adherence to healthy lifestyles with the risk of obesity-related morbidities (ORMs) in individuals with high genetic predisposition (A) The risk of 20 ORMs in highly genetically predisposed individuals (top 20% PGS_BMI_ versus remaining 80% PGS_BMI_); adjusted hazard ratios were obtained. Our analysis was deemed to have replicated known causal relationships if the associations exhibited a *p* value below the nominal threshold, and 15 of 20 ORMs reached this threshold. Those replicated in our cohort were used for the subsequent subgroup analysis. Although replicated, associations with coronary artery disease and venous thromboembolism did not reach a Bonferroni threshold (0.05/20), thus warranting cautious interpretations. (B) Subgroup analysis of healthy and unfavorable lifestyles in high PGS_BMI_ carriers for replicated ORMs. An unfavorable lifestyle was defined as a composite of poor and intermediate lifestyles. HR, hazard ratio; CI, confidence interval.

**Figure 5.**
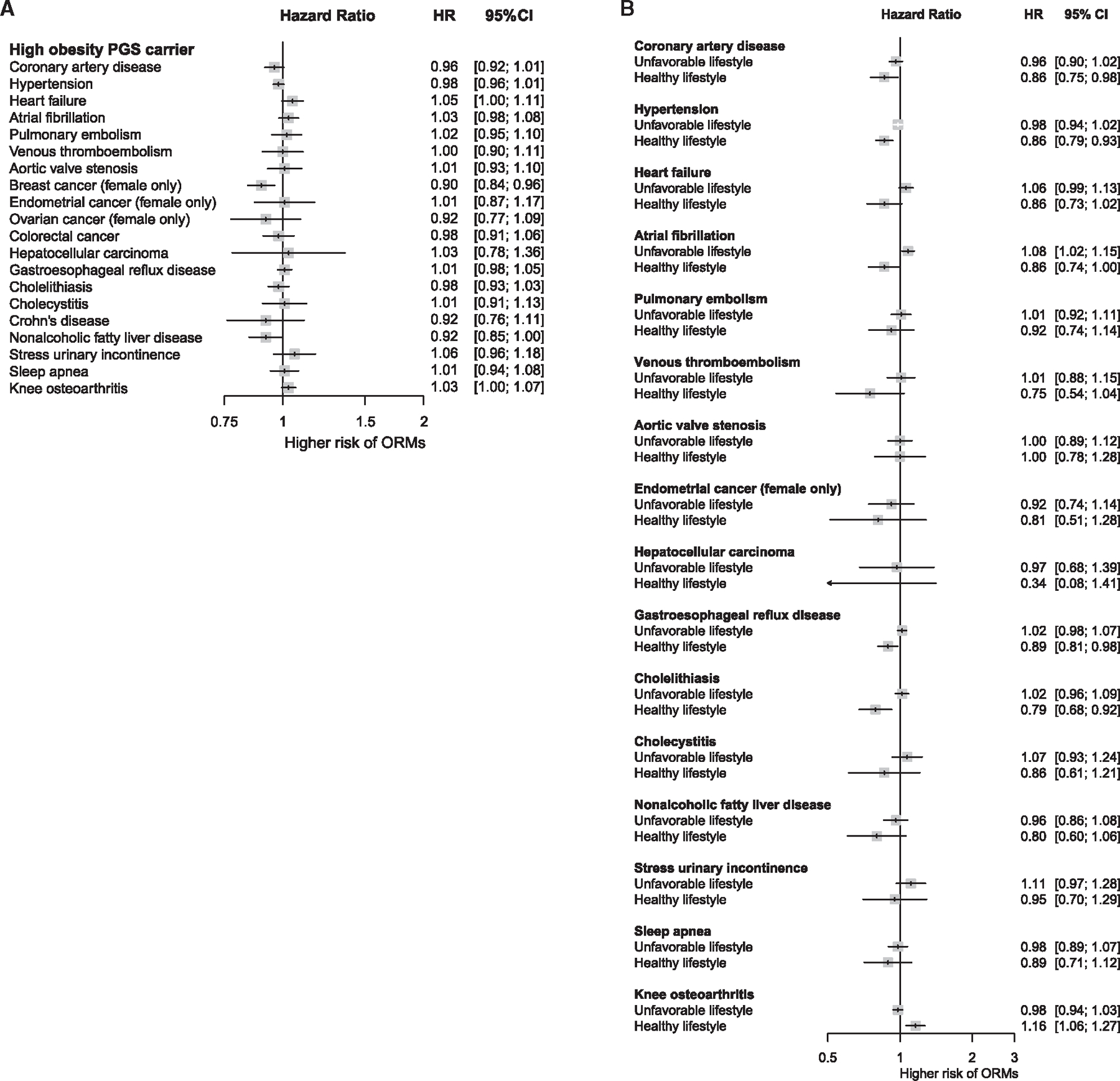
Association of adherence to healthy lifestyles with the risk of obesity-related morbidities (ORMs) in individuals with high genetic predisposition after adjusting for measured BMI (A) The risk of 20 ORMs in highly genetically predisposed individuals (top 20% PGS_BMI_ versus remaining 80% PGS_BMI_); adjusted hazard ratios were obtained. (B) Subgroup analysis of healthy and unfavorable lifestyles in high PGS_BMI_ carriers for ORMs; unfavorable lifestyle was defined as a composite of poor and intermediate lifestyles. HR, hazard ratio; CI, confidence interval.

Our analysis was deemed to have replicated previous findings if the associations possessed a *p* value below the nominal threshold, and 15 of 20 ORMs reached this threshold, thus recapitulating most of the known causal relationships with increased adiposity (Figure 4A). Although replicated, the associations with coronary artery disease and venous thromboembolism did not reach the Bonferroni threshold (0.05/20), thus warranting cautious interpretations. Subgroup analyses according to lifestyle in high PGS_BMI_ carriers revealed that the risks for nearly all ORMs diverged by lifestyle. Those with a high PGS_BMI_ (top 20%) and healthy lifestyle experienced comparable risks of ORM to individuals with non-high PGS_BMI_ (remaining 80%—reference group), whereas those with a high PGS_BMI_ and unfavorable lifestyle showed increased risks of ORMs (Figure 4B). This could be attributed to the smaller BMI differences between the high PGS_BMI_-healthy lifestyle group and the reference group (Table S12). When adjusting for BMI, the association between PGS_BMI_ and the risk of ORMs was null for most conditions (Figure 5), indicating that measured BMI was a strong mediator between PGS_BMI_ and ORMs.

### Weighted lifestyle score

Given that each lifestyle factor composing lifestyle score has a different extent of association with obesity, we constructed a weighted lifestyle score as described in the STAR Methods. The same pattern of associations was observed in a series of sensitivity analyses with the weighted lifestyle score compared to unweighted lifestyle score, as demonstrated in Figure S4 and Table S13.

## DISCUSSION

To the best of our knowledge, this is the largest quantitative analysis of the interplay between genetic and lifestyle risk factors for obesity. Adherence to a healthy lifestyle (minimal obesogenic behaviors) is associated with a reduced risk of obesity and ORMs across all genetic backgrounds. Absolute differences in obesity risk between those who adhere to healthy lifestyles and those who do not had gradually expanded with an increase in polygenic score.

G×E interaction analyses for obesity remain challenging owing to the requirement for a comprehensive cohort with genetic and lifestyle data and a large sample size. Thus far, only 12 loci (e.g., *FTO* and *PPARG*) have been identified to be attenuated or exacerbated by lifestyle factors.^7^ Such focus on selective obesogenic variants of the main effect limits the clinical and public implications because the impact of the variants on body weight change is relatively small (~1 kg) and, subsequently, the attenuation by healthy lifestyles.^10,14^ To extend the variance explained by the genetic predisposition, we leveraged genome-wide variants, including 986,323 common variants, to calculate PGS_BMI_ (aggregating all eligible variants into a single numerical measure); according to our previous study, the collective effects of genome-wide variants could influence a relatively large (~13 kg) gradient in body weight.^8^

We observed that inherited genetic risk and a modifiable lifestyle were both independently and jointly associated with obesity. Our multiplicative interaction analysis yielded consistent results and revealed significant interactions between genetic and individual lifestyle factors, as well as composite lifestyles (*p*_interaction_ < 0.001 for all interactions) (Tables S4–S9). The RERI was also significant (Table S2), indicating the overall risk was greater than the added risk associated with each individual factor. The significant additive interaction may indicate that the public health consequences of a poor lifestyle are greater in populations with high genetic risk. Such interactions between genetic and lifestyle factors may explain the separation of risk trajectories between individuals with healthy lifestyles and those without as genetic scores increased (Figure S3). Current evidence suggests that genetic testing cannot motivate individuals to improve their lifestyle, and some individuals may even worsen their habits after receiving genetic risk information.^15^ This observation may be attributable to the deterministic view that genetic risk is unmodifiable.^9^ Convincing evidence of genetics-by-lifestyle interactions described in our study, however, counters the deterministic views and might give people a sense of control and motivate people to carry out behavioral changes toward weight change. Our findings are particularly relevant to evoking health-giving behaviors in response to a genetic test and gearing people up to maintain a balanced attitude toward precision medicine. Nonetheless, whether the provision of indeterministic evidence can improve obesity and obesity-related outcomes remains to be determined through a prospective study.

Among the diverse lifestyle factors, sedentary behavior (television viewing and computer playing) was most substantially associated with an increased risk of obesity, and this was followed by low physical activity and a suboptimal diet after adjusting for genetic risks. These findings suggest that mitigating sedentary behaviors could be an effective strategy to tackle obesity, given that interventions targeting a sedentary lifestyle would concurrently impact both energy expenditure and diet, contributing to weight management. Television watching appears to encourage snacking and also influences food choices after viewing.^16,17^ Identifying specific factors is clinically crucial, as it can be challenging for people with obesity to simultaneously modify all aspects of their lifestyle. Instead, focused behavior modification aggregates motivations and inputs to comply with lifestyle modification prescriptions.

Adiposity is causally associated with an increased risk of various diseases and has been proposed as an important target for multimorbidity prevention.^3,4^ Although lifestyle modification has long been conceived as a major weight management tactic, its effect on reducing obesity-related traits in genetically predisposed individuals has not yet been assessed. By demonstrating the mitigating effects of healthy lifestyles on multiple obesity-related health outcomes in a high-risk genetic group, this study provides the first empirical evidence of the association between adherence to a healthy lifestyle and multimorbidity prevention in genetically predisposed individuals. This finding implicates potentially huge benefits to healthcare economics with the promotion of a healthy lifestyle given the considerable out-of-pocket expenditure associated with major ORMs such as cardiovascular diseases (CVDs) and cancers.^18–21^

A recent study revealed that PGS for blood pressure predicts the risk of CVDs independent of measured blood pressure, thereby enabling early and targeted CVD prevention.^22^ Furthermore, Cho et al. demonstrated that individuals with high PGS for blood pressure experience an increased risk for CVD, even when their blood pressure is within normal ranges.^22^ We assessed if this holds for PGS_BMI_ by further adjusting for BMI and observed that the association between PGS_BMI_ and the risk of ORM was null for most conditions (Figure 5), thus indicating a strong mediating effect of measured BMI between PGS_BMI_ and the risk of ORMs. Moreover, the high genetic risk group that exhibited BMI levels similar to those in the reference group (not at high genetic risk) faced comparable risks for ORMs, whereas those within the high genetic risk group who possessed higher BMI levels—likely due to poor lifestyles—were observed to have an elevated risk for ORMs (Table S12). This finding offered reassurance that non-obese high PGS_BMI_ carriers may not experience an increased risk of ORMs. Given that the measured BMI is a strong mediator, the prevention of weight gain in advance by adhering to healthy lifestyles, regardless of genetic background, would help to mitigate multimorbidity. Our findings encourage genetic scoring, as it could screen out those who are not obese but are likely to be obese at a later stage of life and guide them toward a healthy lifestyle.

### Limitations of the study

This study had several limitations. First, we used the diagnostic codes for incident obesity. The ICD code for obesity is frequently assigned to patients manifesting comorbidities or those requiring anti-obesity medication or surgery, thus possibly capturing severe cases of obesity.^23^ Such context may have contributed to the relatively low incidence of obesity in our study. Therefore, our results regarding incident obesity, including the probability of obesity risk by age 75, should be understood and interpreted within this specific context. Additionally, to align with the commonly accepted definition of obesity (BMI ≥ 30 kg/m^2^), we also conducted a subgroup analysis for incident obesity defined by the BMI threshold. This analysis utilized a subset of 21,715 participants with follow-up BMI measurements and complete lifestyle factor data, among whom 1,202 (6.7%) transitioned to a BMI ≥ 30 during the follow-up period. The results were consistent with our overall findings (Figure S4), thus suggesting that our conclusions may apply to individuals with a BMI ≥ 30 kg/m^2^ irrespective of comorbidities. Second, although associations between lifestyle and obesity in a cross-sectional setting are prone to reverse causation bias, we still presented results based on prevalent obesity as a secondary endpoint. This is due to the observation that the genetic risk for obesity exerts its influence early in life and accumulates throughout the course of life.^8,24^ Given that the UK Biobank recruited a middle-aged population (above the age of 40), many individuals with a high genetic risk for obesity may have already been affected before enrollment. Analyzing only post-enrollment incident cases would overlook a significant proportion of cases driven by high genetic risk, thus potentially underestimating the genetic contribution to obesity. Also, the prevalent obesity was used for interaction analysis to fully capture the influence of genetic risk factors. The consistency of our findings across incident and prevalent obesity reinforces the reliability of our observed associations between genetic and lifestyle factors, effectively negating the possibility of reverse causation where obesity could influence lifestyle choices. Third, we used a number of healthy lifestyle factors as a composite lifestyle score (range from 0 to 5), in line with the prior studies.^11,25^ However, each lifestyle factor comprising the lifestyle score exhibited a different extent of association with obesity (Table S10). To account for this, we constructed a sensitivity analysis using weighted lifestyle score by factoring in the weight of each lifestyle factor and used the weighted score for downstream analyses.^26^ The results remained consistent as presented in Figure S4 and Table S13. Last, our analysis was limited to individuals of European ancestry. The generalizability of our findings should be examined in non-European populations as robust and larger ancestry-specific data become available.

### Conclusions

High genetic risk and an obesogenic lifestyle were independently and jointly associated with a high risk of obesity. Adherence to a healthy lifestyle can significantly counteract the genetic predisposition to obesity. Furthermore, adherence to healthy lifestyles that are conducive to weight loss mitigates the risk of obesity-associated morbidities in genetically predisposed individuals. Healthy lifestyles should be promoted irrespective of genetic background.

## STAR★METHODS

### RESOURCE AVAILABILITY

#### Lead contact

Further information and requests for resources should be directed to and will be fulfilled by the lead contact, Hong-Hee Won (wonhh@skku.edu).

#### Materials availability

This study did not generate new reagents.

#### Data and code availability

- The data supporting the findings of this study are available in the following resources. Data from UK Biobank are available on application to its site (https://www.ukbiobank.ac.uk/enable-your-research/apply-for-access). The GWAS summary statistics for body mass index from the GIANT consortium are available on the website (https://portals.broadinstitute.org/collaboration/giant/index.php/GIANT_consortium_data_files#GWAS_Anthropometric_2015_BMI_Summary_Statistics).
- This paper does not report original code.
- Any additional information required to reanalyze the data reported in this paper is available from the lead contact upon request.

### EXPERIMENTAL MODEL AND STUDY PARTICIPANT DETAILS

#### Study populations

The UK Biobank study was approved by the National Information Governance Board for Health and Social Care and the North West Multicentre Research Ethical Committee (11/NW/0382), and all participants provided informed consent to participate.^27^ This research was conducted using the UK Biobank Resource under application number 33002. Individuals with missing or low-quality genetic information, those who were genetically related, or those who withdrew consent were excluded from the analysis. A total of 338,645 unrelated, white-British, and genotype quality-controlled participants with both clinical and genotypic data were included.

### METHOD DETAILS

#### Genotyping and imputation

Details of genotyping and imputation for 487, 409 UK Biobank samples have been described elsewhere.^27^ In brief, genotype calling was performed by either the Affymetrix UK BiLEVE Axiom or UK Biobank Axiom arrays, which have over 95% coverage and include 805,426 variants. Imputation was performed centrally using a combination of UK 10K and 1000 Genomes Phase 3 panels. We considered white British participants based on genetic ethnic grouping data (Data-Field 22006) and excluded samples with a discrepancy between the reported and genetic sex or putative sex chromosome aneuploidy. From the imputed genotype data, we further excluded variants with call rate <95%, Hardy-Weinberg equilibrium *p* < 1 × 10^−6^, imputation quality scores (INFO) < 0.4, or minor allele frequency (MAF) < 0.005. A total of 9,575,249 variants remained after the quality control.

#### Polygenic score

A polygenic score (PGS), aggregating an individual’s load of common genetic variants, was constructed based on a largest genome-wide association study (GWAS) currently accessible to the public for body mass index (BMI) in 322,154 individuals of European ancestry.^28^ We previously validated that PGS for BMI (PGS_BMI_) accurately predicts the risk for obesity and cardiometabolic diseases, justifying the use of BMI as a proxy to quantify genetic risk for obesity.^8^ PRS-CS, a Bayesian polygenic prediction method,^29^ was utilized for training the posterior effect sizes of single nucleotide polymorphisms (SNPs) from the GWAS summary statistics with the external linkage disequilibrium (LD) reference panel constructed using the 1000 Genomes phase 3 European data. We applied PRS-CS-auto, which enables automatic learning of a global scaling parameter from the GWAS results with no requirement for an independent validation dataset; since it was demonstrated that both PRS-CS and PRS-CS-auto outperformed all alternative methods for BMI in the Mass General Brigham Biobank (formerly, Partners HealthCare Biobank).^29,30^ The default settings were applied to all other parameters. PGS_BMI_ were standardized to have a mean of 0 and a standard deviation of 1, and then categorized into low (lowest quintile), intermediate (quintiles 2–4), and high (highest quintile) genetic risk. The PGS_BMI_ demonstrated an association with the measured BMI, and exhibited normal distribution, and the categories of genetic risk were proportionally distributed across lifestyle groups (Figure S1).

#### Healthy lifestyle score

A healthy lifestyle score was constructed from five previously described obesogenic lifestyle factors^16,31^ collected at enrollment, and these included physical activity, diet, sedentary behaviors, alcohol consumption, and sleep duration (Table S14). The specific criteria were determined using previously defined cutoffs and the direction of change in obesity risk.^11,25,32^ A healthy diet pattern was defined as a diet that includes at least half of the following eight criteria: consumption of an increased amount of 1) fruits, 2) vegetables, 3) whole grains, and 4) fish, and a reduced amount of 5) refined grains, 6) processed meats, 7) unprocessed red meats, and 8) sugar-sweetened foods or beverages^16,33^; well-being physical activity was activity level exceeding 3000 metabolic equivalents of task (MET) minutes/week (the top 30% of all UK Biobank participants)^34^; less engagement in sedentary behaviors were defined as time spent watching television or using a personal computer (excluding work) not exceeding 2 h/day^16^; low alcohol consumption was ≤14 g/day for women and ≤28 g/day for men, with the maximum limit reflecting US dietary guidelines^35^; adequate sleep duration was 6–8 h/day.^16^ As demonstrated in earlier studies,^11,25^ participants scored 1 point for each healthy behavior, and lifestyle factor scores ranged from 0 to 5, with higher scores indicating higher adherence to healthy lifestyles. The scores were categorized as healthy (4–5 healthy lifestyle factors), intermediate (2–3 healthy lifestyle factors), and poor (0–1 healthy lifestyle factor) lifestyle.^11,25^ As a sensitivity analysis, we constructed a weighted lifestyle score based on the five lifestyle factors using the following equation: weighted lifestyle score = (b1*factor1 + b2*factor 2 + … + b5*factor 5) * (5/sum of the b coefficients).^26,32^ This weighted score also ranged from 0 to 5 points, and participants were categorized into lifestyle groups in a manner consistent with the grouping derived from the raw lifestyle scores.

#### Assessment of outcomes

Incident obesity was the primary endpoint. Incident obesity was determined through the analysis of the UK Biobank health data that included primary care data, hospital inpatient records, death register entries, and self-reported medical conditions. This comprehensive approach identified individuals experiencing their first occurrence of obesity and those who underwent bariatric surgery as indicated by the OPCS-4 codes (Table S15). The date of diagnosis was determined based on the earliest records from any of the sources listed above. Individuals not included in the case group were classified as controls, with the last follow-up date set as December 31, 2021.

We also explored prevalent obesity as a secondary outcome, and this was defined as BMI ≥30 kg/m^2^ at baseline.^7^ Our rationale for including prevalent obesity stemmed from evidence that obesity PGS manifest their impact early in life.^8,24^ Given that UK Biobank enrollment begins at age 40, a considerable number of individuals with a high genetic predisposition may already be living with obesity before enrollment. As such, limiting our analysis to incident obesity cases after enrollment could potentially overlook these significant proportions, possibly leading to an underestimation of the genetic influence on obesity. Conversely, an analysis on prevalent obesity alone would not be sufficient, as associations between lifestyle and obesity in a cross-sectional setting are prone to reverse causation bias, thus creating difficulty in determining if lifestyle is the cause or effect of obesity. Therefore, we established cohorts for incident and prevalent obesity to conduct parallel analyses and cross-validated our findings in both scenarios to strengthen the robustness of our observations.

In addition to BMI, we also assessed body fat percentage, waist circumference (WC), and waist-to-hip ratio (WHR) at baseline. Top 20% was defined as cutoffs to establish an equivalent point to BMI (BMI of 30 represented the top 20% among UK Biobank participants). We predicted absolute risks by estimating the hazard ratio (HR) and odds ratio (OR) of incident and prevalent obesity, respectively, by lifestyle groups and PGS percentile. Furthermore, we calculated the HRs of obesity using the Cox proportional hazard regression model and ORs of obesity using a logistic regression model with enrollment age, sex, and the first four principal components of ancestry as covariates. The predicted probability of obesity by age 75 was then calculated by referencing the risk of the poor lifestyle group with the mean PGS and conditioning on the mean value of each covariate. The difference in percentage units between the healthy and poor lifestyle groups across the PGS percentiles was then assessed.

Twenty obesity-related morbidities (ORMs) previously shown to be causally affected by increased adiposity in Mendelian randomization or interventional studies were selected for subsequent analysis (Table S16).^4,36,37^ Incident ORMs were ascertained using self-reported, death registry records, and hospital admission data from the UK Biobank. We applied the Bonferroni correction to counteract multiple comparisons problems.

#### Covariates

All models were adjusted for enrollment age, sex, education (college/university degree or professional qualifications [e.g., nursing, teaching] = 1, others = 0), socioeconomic status (categories derived from TDI of the bottom 20% [least deprived], middle 60% [intermediate], and top 20% [most deprived], combining information on social class, employment, car availability, and housing), smoking status, diabetes mellitus, weight gain medications (Table S17), and other weight changing medical conditions (depression, Cushing syndrome, hypothyroidism, and polycystic ovary syndrome).^38^ We further adjusted for the genotype array and the first 10 principal components (PCs) when models included the PGS_BMI_.

#### Multiplicative and additive interactions

For the interaction analysis, we utilized prevalent obesity to fully capture the influence of genetic risk factors accumulated and manifested from pre-enrollment.^12,13^ We evaluated the interaction between lifestyle and genetic risk for obesity using multiplicative and additive interaction analyses, as previously described.^39–41^ Multiplicative interaction was assessed by adding a multiplicative interaction term in the logistic regression models. Additive interaction between genetic and lifestyle factors on the risk of obesity was assessed by using two continuous variables (PGS_BMI_ and lifestyle scores) and examining the relative excess risk due to interaction (RERI) as an index of additive interaction (RERI >0 indicates the presence of significant additive interactions).^42,43^ We further analyzed the decomposition of the joint effect—the proportions attributable (proportion of effect under double exposure) to genetic risk alone, lifestyle alone, and their additive interaction.

### QUANTIFICATION AND STATISTICAL ANALYSIS

The Cox proportional hazard regression models were used to examine the association of genetic risk and lifestyle with incident obesity and ORMs. The first date of visiting the study assessment center (between 2006 and 2010) was considered the index date, and participants were followed up until the date of first diagnosis, death, loss to follow-up, or the end of the follow-up for the current data (December 31, 2021), whichever came first. The Median (interquartile range [IQR]) length of follow-up for 20 ORMs was 11.55 (11.52–11.56) years. Multivariable logistic regression was used to examine the association of genetic risk categories, lifestyle categories, and the combination of genetic and lifestyle categories (nine categories with low genetic risk and healthy lifestyle as reference) with prevalent obesity.

We compared the incident risk of 20 ORMs between high PGS_BMI_ carriers (top 20%) and the remaining 80% PGS_BMI_ carriers (reference group). We then conducted subgroup analyses by separating those adhering to a healthy or unfavorable lifestyle among the high PGS_BMI_ carriers to observe whether adherence to healthy lifestyles could offset the risk of ORMs. To differentiate the effect of PGS_BMI_ from that of BMI, we replicated the ORM analysis by adjusting for BMI. *p* values were two-sided, with statistical significance set at *p* < 0.05. All analyses other than genetic computations were performed using R software (version 3.6.0).

## Supplementary Material

Document S1. Figures S1–S4 and Tables S1–S17

Data S1. Unprocessed source data underlying all graphs and plots, related to Figures 3, S1, and S3

Supplemental information can be found online at https://doi.org/10.1016/j.cmet.2024.06.004.

## Figures and Tables

**Table 1.** Baseline characteristics of participants in this study investigating the association of lifestyle and genetic risk with incident obesity

Characteristics	Non-obese (*n* = 173,672)	Obese (*n* = 4,108)
Sex, no. (%)		
Female	88,314 (50.9)	1,877 (45.7)
Male	85,358 (49.1)	2,231 (54.3)
Age, mean (SD)	57.04 (8.07)	57.95 (7.87)
Genetic risk category, no. (%)		
High (top 20%)	34,502 (19.9)	1,054 (25.7)
Intermediate (middle 60%)	104,176 (60.0)	2,492 (60.7)
Low (bottom 20%)	34,994 (20.1)	562 (13.7)
Lifestyle factors, no. (%)		
Physical activity:favorable	56,193 (32.4)	1,444 (35.2)
Physical activity: unfavorable	117,479 (67.6)	2,664 (64.8)
Diet: favorable	64,129 (36.9)	1,364 (33.2)
Diet: unfavorable	109,543 (63.1)	2,744 (66.8)
Alcohol consumption: favorable	116,917 (67.3)	2,659 (64.7)
Alcohol consumption: unfavorable	56,755 (32.7)	1,449 (35.3)
Sedentary behavior: favorable	57,660 (33.2)	962 (23.4)
Sedentary behavior: unfavorable	116,012 (66.8)	3,146 (76.6)
Sleep duration: favorable	155,201 (89.4)	3,556 (86.6)
Sleep duration: unfavorable	18,471 (10.6)	552 (13.4)
No. of healthy lifestyle factors, no. (%)		
0	2,162 (1.2)	70 (1.7)
1	22,185 (12.8)	627 (15.3).
2	58,644 (33.8)	1,511 (36.8)
3	57,695 (33.2)	1,338 (32.6)
4	27,388 (15.8)	488 (11.9)
5	5,598 (3.2)	74 (1.8)
Lifestyle category, no (%)		
Poor	24,347 (14.0)	697 (17.0)
Intermediate	116,339 (67.0)	2,849 (69.4)
Healthy	32,986 (19.0)	562 (13.7)
Education, no. (%)		
Higher education	75,221 (43.3)	1,301 (31.7)
Remainder	97,707 (56.3)	2,788 (67.9)
Unknown	744 (0.4)	19 (0.5)
Socioeconomic status quintile, no. (%)		
1 (least deprived)	34,810 (20.0)	706 (17.2)
2 to 4	104,143 (60.0)	2,404 (58.5)
5 (most deprived)	34,520 (19.9)	995 (24.2)
Unknown	199 (0.1)	3 (0.1)
Smoking status, no. (%)		
Never	96,493 (55.6)	1,913 (46.6)
Previous	60,455 (34.8)	1,650 (40.2)
Current	16,365 (9.4)	533 (13.0)
Unknown	359 (0.2)	12 (0.3)
Alcohol drinking status, no. (%)		
Never	5,067 (2.9)	134 (3.3)
Previous	5,585 (3.2)	195 (4.7)
Current	162,948 (93.8)	3,776 (91.9)
Unknown	72 (0.0)	3 (0.1)
Other, no. (%)		
Diabetes	4,382 (2.5)	190 (4.6)
Depression	1,062 (0.6)	59 (1.4)
Cushing’s syndrome	14 (0.0)	0 (0.0)
Hypothyroidism	1,632 (0.9)	68 (1.7)
Polycystic ovary syndrome	37 (0.0)	1 (0.0)
Weight gain medication prescriptions	10,562 (6.1)	397 (9.7)

**Table 2.** The associations of genetic risk and lifestyles with incident obesity

	Model 1^a^			Model 2^b^		

Genetic risk groups	low (*n* = 35,556)	intermediate (*n* = 106,668)	high (*n* = 35,556)	low (*n* = 35,556)	intermediate (*n* = 106,668)	high (*n* = 35,556)
No. of cases	562	2,492	1,054	562	2,492	1,054
HR (95% CI)	Ref.	1.48 (1.35–1.62)	1.85 (1.67–2.06)	Ref.	1.48 (1.35–1.63)	1.86 (1.68–2.07)
*p* value		<0.001	<0.001		<0.001	<0.001
*p* value for trend	<0.001			<0.001		

	Model 1^c^			Model 2^d^		

Lifestyle groups	healthy (*n* = 33,548)	intermediate (*n* = 119,188)	poor (*n* = 25,044)	healthy (*n* = 33,548)	intermediate (*n* = 119,188)	poor (*n* = 25,044)
No. of cases	562	2,849	697	562	2,849	697
HR (95% CI)	Ref.	1.34 (1.22–1.47)	1.47(1.31–1.64)	Ref.	1.35 (1.23–1.48)	1.48 (1.32–1.67)
*p* value		<0.001	<0.001		<0.001	<0.001
*p* value for trend	<0.001			<0.001		

CI, confidence interval; HR, hazard ratio.

a Cox proportional hazard regression adjusted for age, sex, education, socioeconomic status, genotyping arrays, the first 10 principal components of ancestry, smoking status, diabetes, depression, Cushing syndrome, hypothyroidism, polycystic ovary, and weight-gaining medicine use; *p* value for trends was calculated by treating the polygenic score as a continuous variable.

b Isolated effect of genetic risk on incident obesity (independent of lifestyles); Cox proportional hazard regression adjusted for model 1 and lifestyle groups; *p* value for trends was calculated by treating the polygenic score as a continuous variable.

c Cox proportional hazard regression adjusted for age, sex, education, socioeconomic status, genotyping arrays, the first 10 principal components of ancestry, smoking status, diabetes, depression, Cushing syndrome, hypothyroidism, polycystic ovary, and weight-gaining medicine use; *p* value for trends was calculated by treating the lifestyle score as a continuous variable.

d Isolated effect of lifestyles on incident obesity (independent of genetic risk); Cox proportional hazard regression adjusted for model 1 and genetic risk groups; *p* value for trends was calculated by treating the lifestyle score as a continuous variable.

**Table:** KEY RESOURCES TABLE

REAGENT or RESOURCE	SOURCE	IDENTIFIER
Deposited data		
UK Biobank	Bycroft et al.^27^	https://www.ukbiobank.ac.uk/
GWAS summary statistics for body mass index	Locke et al.^28^	https://portals.broadinstitute.org/collaboration/giant/index.php/GIANT_consortium_data_files#GWAS_Anthropometric_2015_BMI_Summary_Statistics
Software and algorithms		
PRS-CS	Ge et al.^29^	https://github.com/getian107/PRScs
R (version 3.6.0)	The R Project for Statistical Computing	https://www.r-project.org/

## Highlights

- High genetic risk and obesogenic lifestyle jointly raise obesity risk
- High genetic predisposition to obesity increases risk of obesity-related morbidities
- Healthy lifestyle prevents morbidities, even in individuals with a high genetic risk
- Healthy lifestyles should be promoted irrespective of genetic background
