# Supplementary material for: Association of genetic risk, lifestyle, and their interaction with obesity and obesity-related morbidities
Source: Cell Metab. Author manuscript; Available in PMC 2025 Jul 23. (PMC12285577; doi:10.1016/j.cmet.2024.06.004)
Supplement: Document S1. Figures S1–S4 and Tables S1–S17 [file NIHMS2091572-supplement-Document_S1__Figures_S1_S4_and_Tables_S1_S17.pdf]

Cell Metabolism, Volume *36*

Supplemental information

Association of genetic risk, lifestyle, and their interaction with obesity and obesity-related morbidities

Min Seo Kim, Injeong Shim, Akl C. Fahed, Ron Do, Woong-Yang Park, Pradeep Natarajan, Amit V. Khera, and Hong-Hee Won

Table S1. Baseline characteristics of participants in this study investigating the association of lifestyle and genetic risk with prevalent obesity. Related to Figure 1.

**Characteristics**	**Non-obese (N=256,035)**	**Obese (N=81,519)**
Sex, no. (%)		
Female	139334 (54.4)	42019 (51.5)
Male	116701 (45.6)	39500 (48.5)
Age, mean (SD)	57.26 (8.06)	57.70 (7.77)
Genetic risk category, no. (%)		
High (top 20%)	41354 (16.2)	26128 (32.1)
Intermediate (middle 60%)	155102 (60.6)	47436 (58.2)
Low (bottom 20%)	59579 (23.3)	7955 (9.8)
Lifestyle factors, no. (%)		
Physical activity		
Favorable	68329 (26.7)	16588 (20.3)
Unfavorable	141837 (55.4)	46869 (57.5)
Unknown	45869 (17.9)	18062 (22.2)
Diet		
Favorable	89399 (34.9)	23614 (29.0)
Unfavorable	156991 (61.3)	53820 (66.0)
Unknown	9645 (3.8)	4085 (5.0)
Alcohol consumption		
Favorable	151664 (59.2)	45469 (55.8)
Unfavorable	71486 (27.9)	20956 (25.7)
Unknown	32885 (12.8)	15094 (18.5)
Sedentary behavior		
Favorable	81146 (31.7)	14606 (17.9)
Unfavorable	172887 (67.5)	65971 (80.9)
Unknown	2002 (0.8)	942 (1.2)
Sleep duration		
Favorable	225411 (88.0)	67689 (83.0)
Unfavorable	29456 (11.5)	13196 (16.2)
Unknown	1168 (0.5)	634 (0.8)
No. of healthy lifestyle factors, no. (%)		
0	2261 (0.9)	1128 (1.4)
1	23100 (9.0)	9599 (11.8)
2	60844 (23.8)	20135 (24.7)
3	59688 (23.3)	14336 (17.6)

4	28132 (11.0)	4636 (5.7)
5	5730 (2.2)	617 (0.8)
Unknown	76280 (29.8)	31068 (38.1)
Lifestyle category, no (%)		
Poor	25361 (9.9)	10727 (13.2)
Intermediate	120532 (47.1)	34471 (42.3)
Healthy	33862 (13.2)	5253 (6.4)
Unknown	76280 (29.8)	31068 (38.1)
Education, no. (%)		
Higher education	100251 (39.2)	23769 (29.2)
Remainder	153593 (60.0)	56814 (69.7)
Unknown	2191 (0.9)	936 (1.1)
Socioeconomic status quintile, no. (%)		
1 (least deprived)	58421 (22.8)	14790 (18.1)
2 to 4	157955 (61.7)	48866 (59.9)
5 (most deprived)	39365 (15.4)	17750 (21.8)
Unknown	294 (0.1)	113 (0.1)
Smoking status, no. (%)		
Never	143145 (55.9)	40972 (50.3)
Previous	85895 (33.5)	32558 (39.9)
Current	26192 (10.2)	7617 (9.3)
Unknown	803 (0.3)	372 (0.5)
Alcohol drinking status, no. (%)		
Never	7196 (2.8)	3186 (3.9)
Previous	7847 (3.1)	3656 (4.5)
Current	240785 (94.0)	74597 (91.5)
Unknown	207 (0.1)	80 (0.1)
Diabetes, no. (%)	7137 (2.8)	8948 (11.0)
Depression, no. (%)	1827 (0.7)	1123 (1.4)
Cushing's syndrome, no. (%)	18 (0.0)	25 (0.0)
Hypothyroidism, no. (%)	2768 (1.1)	1739 (2.1)
Polycystic ovary syndrome, no. (%)	60 (0.0)	51 (0.1)
Weight gain medication prescriptions, no. (%)	17373 (6.8)	12172 (14.9)

Table S2. The associations of genetic risk and lifestyles, and their interaction with prevalent obesity. Related to Table 2.

	**Model 1[a]**			**Model 2[b]**		
**Genetic risk groups**	Low (n = 67,534)	Intermediate (n = 202,538)	High (n = 67,482)	Low (n = 67,534)	Intermediate (n = 202,538)	High (n = 67,482)
**No. of cases**	7,955	47,436	26,128	7,955	47,436	26,128
**OR (95% CI)**	Reference	2.26 (2.20-2.32)	4.61 (4.47-4.74)	Reference	2.25 (2.17-2.32)	4.59 (4.43-4.76)
P value		< 0.001	< 0.001		< 0.001	< 0.001
P value for trend	< 0.001			< 0.001		
	**Model 1[c]**			**Model 2[d]**		
**Lifestyle groups**	Healthy (n = 39,115)	Intermediate (n = 155,003)	Poor (n = 36,088)	Healthy (n = 39,115)	Intermediate (n = 155,003)	Poor (n = 36,088)
**No. of cases**	5,253	34,471	10,727	5,253	34,471	10,727
**OR (95% CI)**	Reference	1.76 (1.70-1.82)	2.47 (2.38-2.57)	Reference	1.79 (1.73-1.85)	2.57 (2.47-2.67)
P value		< 0.001	< 0.001		< 0.001	< 0.001
P value for trend	< 0.001			< 0.001		
**Additive interaction**						
Relative excess risk due to interaction (RERI)*				0.25 (0.23-0.26)		
**Attributable proportion, % (95% CI)**						
Genetic risk alone				56.62 (54.27-58.98)		
Lifestyle alone				25.24 (23.90-26.59)		
Additive interaction				18.13 (17.05-19.22)		

Abbreviations: CI, confidence interval; OR, odds ratio
*RERI > 0 indicates additive interactions

[a] Multivariable logistic regression adjusted for age, sex, education, socioeconomic status, genotyping arrays, the first 10 principal components of ancestry, smoking status, diabetes, depression, Cushing syndrome, hypothyroidism, polycystic ovary, and weight-gaining medicine use; P value for trend calculated treating the polygenic score as a continuous variable
[b] Isolated effect of genetic risk on obesity (independent of lifestyles); multivariable logistic regression adjusted for model 1 and lifestyle groups; P value for trend calculated treating the polygenic score as a continuous variable
[c] Multivariable logistic regression adjusted for age, sex, education, socioeconomic status, genotyping arrays, the first 10 principal components of ancestry, smoking status, diabetes, depression, Cushing

syndrome, hypothyroidism, polycystic ovary, and weight-gaining medicine use; P value for trend calculated treating the lifestyle score as a continuous variable

[d] Isolated effect of lifestyles on obesity (independent of genetic risk); multivariable logistic regression adjusted for model 1 and genetic risk groups; P value for trend calculated treating the lifestyle score as a continuous variable

Table S3. Risk of prevalent and incident obesity according to number of healthy lifestyle factors. Related to Table 2.

**Risk of prevalent obesity according to number of healthy lifestyle factors**				
**Lifestyle risk category**	**No. of samples**	**OR[a]**	**95% CI**	**P value**
5 healthy lifestyle factors	6354	0.23	0.21-0.26	< 2e-16
4 healthy lifestyle factors	32831	0.35	0.32-0.38	< 2e-16
3 healthy lifestyle factors	74215	0.51	0.47-0.55	< 2e-16
2 healthy lifestyle factors	81241	0.69	0.64-0.75	< 2e-16
1 healthy lifestyle factors	32814	0.86	0.79-0.93	0.0002
0 healthy lifestyle factors	3408	1 [reference]		
P for trend	< 2e-16			
**Risk of incident obesity according to number of healthy lifestyle factors**				
**Lifestyle risk category**	**No. of samples**	**HR[b]**	**95% CI**	**P value**
5 healthy lifestyle factors	5672	0.53	0.38-0.73	0.0002
4 healthy lifestyle factors	27876	0.66	0.51-0.86	0.0019
3 healthy lifestyle factors	59033	0.83	0.65-1.07	0.1451
2 healthy lifestyle factors	60155	0.90	0.70-1.15	0.3995
1 healthy lifestyle factors	22812	0.95	0.74-1.23	0.7151
0 healthy lifestyle factors	2232	1 [reference]		
P for trend	6.67E-14			

[a]The odds ratio (OR) indicates the risk of obesity according to the number of healthy lifestyles. Multivariable logistic regression adjusted for the polygenic score for BMI, age, sex, education, socioeconomic status, genotyping arrays, PC1 to 10, smoking status, diabetes, depression, Cushing syndrome, hypothyroidism, polycystic ovary, and weight-gaining medicine use.

[b]The hazard ratio (HR) indicates the risk of obesity according to the number of healthy lifestyles. Cox regression adjusted for polygenic score for BMI, age, sex, education, socioeconomic status, genotyping arrays, PC1 to 10, smoking status, diabetes, depression, Cushing syndrome, hypothyroidism, polycystic ovary, and weight-gaining medicine use.

Table S4. Multiplicative interaction between composite lifestyle and genetic risk on BMI. Related to Table 2.

	**Beta**	**SE**	**t value**	**P value**
BMI polygenic score	0.8824	0.0302	29.1924	< 2e-16
Composite lifestyle score (poor)	0.6495	0.0085	76.5515	< 2e-16
Age	0.0083	0.0011	7.4537	9.11E-14
Sex (male)	0.8754	0.0175	50.0147	< 2e-16
Higher education	-0.7352	0.0178	-41.3588	< 2e-16
PC1	-0.0175	0.0056	-3.1025	1.92E-03
PC2	-0.0028	0.0059	-0.4690	6.39E-01
PC3	-0.0017	0.0057	-0.2972	7.66E-01
PC4	0.0145	0.0042	3.4362	5.90E-04
PC5	0.0098	0.0019	5.2271	1.72E-07
PC6	-0.0068	0.0054	-1.2522	2.10E-01
PC7	0.0114	0.0048	2.3512	1.87E-02
PC8	-0.0173	0.0048	-3.6140	3.02E-04
PC9	-0.0064	0.0019	-3.3220	8.94E-04
PC10	0.0000	0.0042	-0.0071	9.94E-01
TDI quintile 2 to 4	0.2488	0.0212	11.7141	< 2e-16
TDI quintile 5	0.6950	0.0290	23.9229	< 2e-16
Previous smoker	0.3470	0.0189	18.3919	< 2e-16
Current smoker	-0.7360	0.0312	-23.5822	< 2e-16
Diabetes	2.7721	0.0454	61.0593	< 2e-16
Depression	0.8903	0.0991	8.9816	< 2e-16
Cushing syndrome	1.8299	0.8249	2.2185	2.65E-02
Hypothyroidism	1.3471	0.0812	16.5850	< 2e-16
Polycystic ovary	3.3442	0.5004	6.6829	2.35E-11
Weight-gaining medicine use	1.1675	0.0341	34.2290	< 2e-16
**BMI PGS*Lifestyle**	**0.0861**	**0.0083**	**10.3500**	**< 2e-16**

Table S5. Multiplicative interaction between physical activity and genetic risk on BMI. Related to Table 2.

	**Beta**	**SE**	**t value**	**P value**
BMI polygenic score	1.0343	0.0155	66.5863	< 2e-16
Less physical activity	0.6971	0.0187	37.1900	< 2e-16
Age	0.0013	0.0011	1.1298	2.59E-01
Sex (male)	0.8316	0.0176	47.2296	< 2e-16
Higher education	-0.6637	0.0179	-36.9803	< 2e-16
PC1	-0.0180	0.0056	-3.2099	1.33E-03
PC2	-0.0018	0.0058	-0.3068	7.59E-01
PC3	-0.0022	0.0056	-0.3878	6.98E-01
PC4	0.0152	0.0042	3.6155	3.00E-04
PC5	0.0106	0.0019	5.6981	1.21E-08
PC6	-0.0072	0.0054	-1.3388	1.81E-01
PC7	0.0108	0.0048	2.2481	2.46E-02
PC8	-0.0173	0.0048	-3.6230	2.91E-04
PC9	-0.0053	0.0019	-2.7506	5.95E-03
PC10	0.0018	0.0042	0.4277	6.69E-01
TDI quintile 2 to 4	0.2501	0.0211	11.8285	< 2e-16
TDI quintile 5	0.6926	0.0289	23.9333	< 2e-16
Previous smoker	0.4190	0.0190	22.0992	< 2e-16
Current smoker	-0.6291	0.0312	-20.1437	< 2e-16
Diabetes	2.6990	0.0453	59.6292	< 2e-16
Depression	0.8437	0.0987	8.5457	< 2e-16
Cushing syndrome	1.6766	0.8210	2.0421	4.11E-02
Hypothyroidism	1.2834	0.0809	15.8694	< 2e-16
Polycystic ovary	3.2562	0.4981	6.5375	6.27E-11
Weight-gaining medicine use	1.1290	0.0340	33.2082	< 2e-16
Unhealthy diet	0.5743	0.0185	31.0860	< 2e-16
More alcohol consumption	0.0697	0.0187	3.7267	1.94E-04
Sedentary behavior	1.3073	0.0193	67.5863	< 2e-16
Unhealthy sleep duration	0.5152	0.0271	19.0410	< 2e-16
**BMI PGS*Physical activity**	**0.1949**	**0.0187**	**10.4481**	**< 2e-16**

Table S6. Multiplicative interaction between diet and genetic risk on BMI. Related to Table 2.

	**Beta**	**SE**	**t value**	**P value**
BMI polygenic score	1.0831	0.0145	74.7634	< 2e-16
Unhealthy diet	0.5753	0.0185	31.1367	< 2e-16
Age	0.0013	0.0011	1.1919	2.33E-01
Sex (male)	0.8300	0.0176	47.1376	< 2e-16
Higher education	-0.6649	0.0179	-37.0429	< 2e-16
PC1	-0.0180	0.0056	-3.2070	1.34E-03
PC2	-0.0016	0.0058	-0.2758	7.83E-01
PC3	-0.0021	0.0056	-0.3790	7.05E-01
PC4	0.0153	0.0042	3.6419	2.71E-04
PC5	0.0107	0.0019	5.7292	1.01E-08
PC6	-0.0075	0.0054	-1.3881	1.65E-01
PC7	0.0110	0.0048	2.2926	2.19E-02
PC8	-0.0171	0.0048	-3.5874	3.34E-04
PC9	-0.0052	0.0019	-2.7242	6.45E-03
PC10	0.0019	0.0042	0.4603	6.45E-01
TDI quintile 2 to 4	0.2503	0.0211	11.8364	< 2e-16
TDI quintile 5	0.6915	0.0289	23.8944	< 2e-16
Previous smoker	0.4184	0.0190	22.0651	< 2e-16
Current smoker	-0.6309	0.0312	-20.1986	< 2e-16
Diabetes	2.7066	0.0453	59.7901	< 2e-16
Depression	0.8422	0.0987	8.5290	< 2e-16
Cushing syndrome	1.6770	0.8211	2.0423	4.11E-02
Hypothyroidism	1.2842	0.0809	15.8765	< 2e-16
Polycystic ovary	3.2484	0.4981	6.5210	7.00E-11
Weight-gaining medicine use	1.1295	0.0340	33.2195	< 2e-16
Less physical activity	0.6949	0.0187	37.0739	< 2e-16
More alcohol consumption	0.0701	0.0187	3.7486	1.78E-04
Sedentary behavior	1.3085	0.0193	67.6414	< 2e-16
Unhealthy sleep duration	0.5166	0.0271	19.0926	< 2e-16
**BMI PGS*Diet**	**0.1332**	**0.0180**	**7.4003**	**1.36E-13**

Table S7. Multiplicative interaction between alcohol consumption and genetic risk on BMI. Related to Table 2.

	**Beta**	**SE**	**t value**	**P value**
BMI polygenic score	1.2221	0.0105	116.1855	< 2e-16
Alcohol consumption	0.0659	0.0187	3.5235	4.26E-04
Age	0.0013	0.0011	1.1526	2.49E-01
Sex (male)	0.8307	0.0176	47.1750	< 2e-16
Higher education	-0.6649	0.0179	-37.0423	< 2e-16
PC1	-0.0180	0.0056	-3.2027	1.36E-03
PC2	-0.0017	0.0058	-0.2869	7.74E-01
PC3	-0.0022	0.0056	-0.3917	6.95E-01
PC4	0.0151	0.0042	3.5989	3.20E-04
PC5	0.0107	0.0019	5.7346	9.79E-09
PC6	-0.0072	0.0054	-1.3442	1.79E-01
PC7	0.0108	0.0048	2.2452	2.48E-02
PC8	-0.0172	0.0048	-3.6070	3.10E-04
PC9	-0.0053	0.0019	-2.7449	6.05E-03
PC10	0.0019	0.0042	0.4465	6.55E-01
TDI quintile 2 to 4	0.2503	0.0211	11.8365	< 2e-16
TDI quintile 5	0.6916	0.0289	23.8996	< 2e-16
Previous smoker	0.4168	0.0190	21.9830	< 2e-16
Current smoker	-0.6308	0.0312	-20.1937	< 2e-16
Diabetes	2.6996	0.0453	59.6383	< 2e-16
Depression	0.8465	0.0987	8.5736	< 2e-16
Cushing syndrome	1.6861	0.8211	2.0535	4.00E-02
Hypothyroidism	1.2838	0.0809	15.8726	< 2e-16
Polycystic ovary	3.2297	0.4981	6.4838	8.96E-11
Weight-gaining medicine use	1.1287	0.0340	33.1964	< 2e-16
Less physical activity	0.6944	0.0187	37.0474	< 2e-16
Unhealthy diet	0.5736	0.0185	31.0479	< 2e-16
Sedentary behavior	1.3065	0.0193	67.5390	< 2e-16
Unhealthy sleep duration	0.5158	0.0271	19.0646	< 2e-16
**BMI PGS*Alcohol consumption**	**-0.1619**	**0.0184**	**-8.8185**	**< 2e-16**

Table S8. Multiplicative interaction between sedentary behaviors and genetic risk on BMI. Related to Table 2.

	**Beta**	**SE**	**t value**	**P value**
BMI polygenic score	1.0457	0.0157	66.4593	< 2e-16
Sedentary behavior	1.3178	0.0194	68.0296	< 2e-16
Age	0.0013	0.0011	1.1674	2.43E-01
Sex (male)	0.8299	0.0176	47.1316	< 2e-16
Higher education	-0.6657	0.0179	-37.0884	< 2e-16
PC1	-0.0180	0.0056	-3.2153	1.30E-03
PC2	-0.0016	0.0058	-0.2755	7.83E-01
PC3	-0.0023	0.0056	-0.4041	6.86E-01
PC4	0.0153	0.0042	3.6314	2.82E-04
PC5	0.0106	0.0019	5.7139	1.11E-08
PC6	-0.0073	0.0054	-1.3506	1.77E-01
PC7	0.0109	0.0048	2.2717	2.31E-02
PC8	-0.0172	0.0048	-3.6121	3.04E-04
PC9	-0.0052	0.0019	-2.7089	6.75E-03
PC10	0.0019	0.0042	0.4520	6.51E-01
TDI quintile 2 to 4	0.2499	0.0211	11.8201	< 2e-16
TDI quintile 5	0.6906	0.0289	23.8639	< 2e-16
Previous smoker	0.4179	0.0190	22.0397	< 2e-16
Current smoker	-0.6299	0.0312	-20.1656	< 2e-16
Diabetes	2.6980	0.0453	59.6018	< 2e-16
Depression	0.8453	0.0987	8.5614	< 2e-16
Cushing syndrome	1.6917	0.8211	2.0603	3.94E-02
Hypothyroidism	1.2853	0.0809	15.8920	< 2e-16
Polycystic ovary	3.2585	0.4981	6.5416	6.10E-11
Weight-gaining medicine use	1.1287	0.0340	33.1968	< 2e-16
Less physical activity	0.6936	0.0187	37.0046	< 2e-16
Unhealthy diet	0.5742	0.0185	31.0821	< 2e-16
More alcohol consumption	0.0715	0.0187	3.8256	1.30E-04
Unhealthy sleep duration	0.5161	0.0271	19.0755	< 2e-16
**BMI PGS*Sedentary behavior**	**0.1766**	**0.0188**	**9.3934**	**< 2e-16**

Table S9. Multiplicative interaction between sleep duration and genetic risk on BMI. Related to Table 2.

	**Beta**	**SE**	**t value**	**P value**
BMI polygenic score	1.1430	0.0092	124.3608	< 2e-16
Unhealthy sleep duration	0.5164	0.0271	19.0837	< 2e-16
Age	0.0013	0.0011	1.1879	2.35E-01
Sex (male)	0.8308	0.0176	47.1837	< 2e-16
Higher education	-0.6647	0.0179	-37.0355	< 2e-16
PC1	-0.0180	0.0056	-3.2022	1.36E-03
PC2	-0.0013	0.0058	-0.2297	8.18E-01
PC3	-0.0021	0.0056	-0.3689	7.12E-01
PC4	0.0153	0.0042	3.6343	2.79E-04
PC5	0.0106	0.0019	5.7171	1.08E-08
PC6	-0.0074	0.0054	-1.3739	1.69E-01
PC7	0.0110	0.0048	2.2746	2.29E-02
PC8	-0.0172	0.0048	-3.6029	3.15E-04
PC9	-0.0053	0.0019	-2.7583	5.81E-03
PC10	0.0019	0.0042	0.4542	6.50E-01
TDI quintile 2 to 4	0.2496	0.0211	11.8037	< 2e-16
TDI quintile 5	0.6906	0.0289	23.8639	< 2e-16
Previous smoker	0.4181	0.0190	22.0481	< 2e-16
Current smoker	-0.6299	0.0312	-20.1658	< 2e-16
Diabetes	2.7000	0.0453	59.6472	< 2e-16
Depression	0.8443	0.0987	8.5512	< 2e-16
Cushing syndrome	1.6857	0.8211	2.0530	4.01E-02
Hypothyroidism	1.2833	0.0809	15.8663	< 2e-16
Polycystic ovary	3.2550	0.4981	6.5344	6.40E-11
Weight-gaining medicine use	1.1292	0.0340	33.2111	< 2e-16
Less physical activity	0.6938	0.0187	37.0131	< 2e-16
Unhealthy diet	0.5745	0.0185	31.0949	< 2e-16
More alcohol consumption	0.0701	0.0187	3.7466	1.79E-04
Sedentary behavior	1.3085	0.0193	67.6439	< 2e-16
**BMI PGS*Sleep duration**	**0.2237**	**0.0268**	**8.3567**	**< 2e-16**

Table S10. Association of lifestyle factors with prevalent obesity stratified by genetic risk. Related to Table 2.

Lifestyle factors	Low genetic risk			Intermediate genetic risk			High genetic risk		
	OR	95% CI	P value	OR	95% CI	P value	OR	95% CI	P value
Physical activity	0.70	0.65-0.74	< 2e-16	0.70	0.68-0.72	< 2e-16	0.70	0.68-0.73	< 2e-16
Diet	0.76	0.72-0.81	< 2e-16	0.73	0.72-0.75	< 2e-16	0.73	0.70-0.76	< 2e-16
Alcohol consumption	0.91	0.86-0.97	0.0018	0.99	0.97-1.02	0.5696	1.06	1.02-1.10	0.0027
Sedentary behavior	0.49	0.46-0.52	< 2e-16	0.53	0.52-0.55	< 2e-16	0.54	0.51-0.56	< 2e-16
Sleep duration	0.71	0.67-0.76	< 2e-16	0.78	0.76-0.81	< 2e-16	0.75	0.72-0.79	< 2e-16

The odds ratio (OR) indicates the risk of obesity for adherence to healthy lifestyle factors. Multivariable logistic regression adjusted for age, sex, education, socioeconomic status, genotyping arrays, PC1 to 10, smoking status, diabetes, depression, Cushing syndrome, hypothyroidism, polycystic ovary, and weight-gaining medicine use.

Table S11. Additive interaction and attributed proportions of genetic risk and each lifestyle factor for prevalent obesity. Related to Table 2.

Lifestyle factors	Attributable proportion (%)	95% CI (%)
Physical activity	28.11	26.60-29.62
Diet	24.80	23.19-26.42
Alcohol consumption	4.87	1.71-8.03
Sedentary behavior	39.45	38.33-40.57
Sleep duration	23.20	20.57-25.83

Table S12. BMI estimates among groups for high PGS_{BMI} carriers with unfavorable lifestyle and high PGS_{BMI} carriers with healthy lifestyle relative to remaining PGS_{BMI} carriers with all lifestyle (reference). Related to Figure 4.

	**Lifestyle group**	**HR**	**Lower CI**	**Upper CI**	**P value**	**Mean BMI**	**SD**	**P value***
**Coronary artery disease**	Unfavorable lifestyle	1.06	1.00	1.12	5.64E-02	29.25	5.17	< 0.001
	Healthy lifestyle	0.89	0.78	1.02	9.10E-02	27.35	4.50	
	Reference	1.00				26.63	4.21	
**Hypertension**	Unfavorable lifestyle	1.16	1.12	1.21	3.68E-16	28.39	4.70	< 0.001
	Healthy lifestyle	0.90	0.83	0.98	1.30E-02	26.73	4.09	
	Reference	1.00				26.06	3.91	
**Heart failure**	Unfavorable lifestyle	1.26	1.18	1.35	5.59E-12	29.31	5.16	< 0.001
	Healthy lifestyle	0.91	0.77	1.07	2.51E-01	27.39	4.50	
	Reference	1.00				26.68	4.22	
**Atrial fibrillation**	Unfavorable lifestyle	1.24	1.16	1.31	6.33E-12	29.30	5.16	< 0.001
	Healthy lifestyle	0.89	0.76	1.03	1.24E-01	27.38	4.50	
	Reference	1.00				26.66	4.21	
**Pulmonary embolism**	Unfavorable lifestyle	1.20	1.09	1.32	1.51E-04	29.31	5.16	< 0.001
	Healthy lifestyle	0.97	0.78	1.21	7.74E-01	27.38	4.49	
	Reference	1.00				26.67	4.21	
**Venous thromboembolism**	Unfavorable lifestyle	1.14	1.00	1.30	4.21E-02	29.27	5.13	< 0.001
	Healthy lifestyle	0.78	0.56	1.08	1.38E-01	27.36	4.48	
	Reference	1.00				26.65	4.20	
**Aortic valve stenosis**	Unfavorable lifestyle	1.17	1.05	1.30	4.82E-03	29.32	5.17	< 0.001
	Healthy lifestyle	1.05	0.82	1.34	7.19E-01	27.40	4.51	
	Reference	1.00				26.68	4.22	
**Endometrial cancer (female only)**	Unfavorable lifestyle	1.25	1.01	1.54	3.81E-02	28.92	5.70	< 0.001
	Healthy lifestyle	0.89	0.56	1.41	6.26E-01	26.91	4.81	

	Reference	1.00				26.10	4.51	
**Hepatocellular carcinoma**	Unfavorable lifestyle	1.21	0.85	1.72	2.80E-01	29.32	5.17	< 0.001
	Healthy lifestyle	0.36	0.09	1.47	1.56E-01	27.40	4.51	
	Reference	1.00				26.68	4.22	
**Gastroesophageal reflux disease**	Unfavorable lifestyle	1.11	1.06	1.16	1.79E-06	29.25	5.16	< 0.001
	Healthy lifestyle	0.92	0.83	1.01	7.42E-02	27.36	4.51	
	Reference	1.00				26.61	4.21	
**Cholelithiasis**	Unfavorable lifestyle	1.27	1.19	1.35	1.31E-13	29.24	5.13	< 0.001
	Healthy lifestyle	0.85	0.73	0.99	4.14E-02	27.34	4.47	
	Reference	1.00				26.64	4.19	
**Cholecystitis**	Unfavorable lifestyle	1.32	1.14	1.51	1.17E-04	29.31	5.16	< 0.001
	Healthy lifestyle	0.91	0.65	1.29	6.09E-01	27.40	4.51	
	Reference	1.00				26.68	4.22	
**Nonalcoholic fatty liver disease**	Unfavorable lifestyle	1.35	1.21	1.50	1.03E-07	29.32	5.17	< 0.001
	Healthy lifestyle	0.90	0.68	1.19	4.64E-01	27.40	4.51	
	Reference	1.00				26.68	4.22	
**Stress urinary incontinence**	Unfavorable lifestyle	1.23	1.07	1.42	3.00E-03	29.31	5.16	< 0.001
	Healthy lifestyle	0.98	0.72	1.33	9.10E-01	27.39	4.51	
	Reference	1.00				26.68	4.22	
**Sleep apnea**	Unfavorable lifestyle	1.59	1.46	1.74	3.76E-25	29.27	5.12	< 0.001
	Healthy lifestyle	1.03	0.82	1.29	8.04E-01	27.38	4.50	
	Reference	1.00				26.66	4.20	
**Knee osteoarthritis**	Unfavorable lifestyle	1.29	1.24	1.35	9.52E-29	29.25	5.14	< 0.001
	Healthy lifestyle	1.26	1.14	1.38	1.69E-06	27.34	4.48	
	Reference	1.00				26.64	4.20	

* Wilcoxon rank sum test

Table S13. The associations of genetic risk and weighted lifestyle scores, and their interaction with incident obesity. Related to Table 2.

	Model 1[a]			Model 2[b]		
**Genetic risk groups**	Low (n=35,556)	Intermediate (n=106,668)	High (n=35,556)	Low (n=35,556)	Intermediate (n=106,668)	High (n=35,556)
**No. of cases**	562	2,492	1,054	562	2,492	1,054
**HR (95% CI)**	Reference	1.48 (1.35-1.62)	1.85 (1.67-2.06)	Reference	1.48 (1.35-1.63)	1.86 (1.68-2.07)
P value		<0.001	<0.001		<0.001	<0.001
P value for trend	< 0.001			< 0.001		
	**Model 1[c]**			**Model 2[d]**		
**Lifestyle groups***	Favorable (n=15,509)	Intermediate (n=143,319)	Poor (n=18,952)	Favorable (n=15,509)	Intermediate (n=143,319)	Poor (n=18,952)
**No. of cases**	290	3,306	512	290	3,306	512
**HR (95% CI)**	Reference	1.22 (1.08-1.38)	1.36 (1.17-1.57)	Reference	1.23 (1.09-1.39)	1.38 (1.19-1.61)
P value		0.002	<0.001		<0.001	<0.001
P value for trend	<0.001			<0.001		

Abbreviations: CI, confidence interval; HR, Hazard ratio.

*We constructed a weighted lifestyle score based on the five lifestyle factors by using the equation: weighted lifestyle score = (b1*factor1 + b2*factor 2 +. . .+ b5*factor 5) * (5/sum of the b coefficients). This weighted score ranges from 0 to 5 points, and participants are categorized into lifestyle groups in a manner consistent with the grouping derived from raw lifestyle scores.

[a] Cox proportional hazard regression adjusted for age, sex, education, socioeconomic status, genotyping arrays, the first 10 principal components of ancestry, smoking status, diabetes, depression, Cushing syndrome, hypothyroidism, polycystic ovary, and weight-gaining medicine use; P value for trend calculated treating the polygenic score as a continuous variable

[b] Isolated effect of genetic risk on incident obesity (independent of lifestyles); Cox proportional hazard regression adjusted for model 1 and lifestyle groups; P value for trend calculated treating the polygenic score as a continuous variable

[c] Cox proportional hazard regression adjusted for age, sex, education, socioeconomic status, genotyping arrays, the first 10 principal components of ancestry, smoking status, diabetes, depression, Cushing syndrome, hypothyroidism, polycystic ovary, and weight-gaining medicine use; P value for trend calculated treating the lifestyle score as a continuous variable

[d] Isolated effect of lifestyles on incident obesity (independent of genetic risk); Cox proportional hazard regression adjusted for model 1 and genetic risk groups; P value for trend calculated treating the lifestyle score as a continuous variable

Table S14. Definition of five lifestyle factors in UK Biobank. Related to STAR Methods.

**Lifestyle factors**	**Definition**
Physical activity	Score 1 if the total MET minutes per week for all activities (Data-Field 22040) are greater than or equal to 3000 minutes per week, otherwise 0.
Diet	Score 1 if more than 4 of the 8 criteria below are met, otherwise 0.<br>1: Fruits ≥ 3 servings/day (Data-Field 1309, 1319)<br>2: Vegetables ≥ 3 servings/day (Data-Field 1289, 1299)<br>3: Whole grains ≥ 3 servings/day (Data-Field 1438, 1448, 1458, 1468)<br>4: Refined grains ≤ 1.5 servings/day (Data-Field 1438, 1448, 1458, 1468)<br>5: Fish ≥ 2 servings/week (Data-Field 1329, 1339)<br>6: Processed meats ≤ 1 serving/week (Data-Field 1349)<br>7: Unprocessed red meats ≤ 1.5 servings/week (Data-Field 1369, 1379, 1389)<br>8: Never eat sugar-sweetened foods or beverages (Data-Field 6144)
Alcohol consumption	Score 1 if up to 1 drink/day (= 14g/day) for women and up to 2 drinks/day (= 28g/day) for men, otherwise 0.<br>Never alcohol drinker (Data-Field 20117 / Coding: 0, Data-Field 1558 / Coding: 6): 1<br>For those intake alcohol more than once a week (Data-Field 1558 / Coding:1,2,3):<br>- 125ml wine = 0.85 drink equivalents (Data-Field 1568, 1578)<br>- 4% ABV pint beer = 1.28 drink-equivalents (Data-Field 1588)<br>- 25ml spirits = 0.57 drink-equivalents (Data-Field 1598)<br>- 50ml fortified wine = 0.56 drink-equivalents (Data-Field 1608)<br>For those intake alcohol occasionally (Data-Field 1558 / Coding: 4,5):<br>- 125ml wine = 0.85 drink equivalents (Data-Field 4407, 4418)<br>- 4% ABV pint beer = 1.28 drink-equivalents (Data-Field 4429)<br>- 25ml spirits = 0.57 drink-equivalents (Data-Field 4440)<br>- 50ml fortified wine = 0.56 drink-equivalents (Data-Field 4451)<br>(1 drink-equivalent described as containing 14g of pure alcohol.)
Sedentary behavior	Score 1 if the sum of the hours below is less than 2, otherwise 0.<br>1: Time spent watching television (TV) (Data-Field 1070)<br>2: Time spent using computer (not including computer use at work) (Data-Field 1080)
Sleep duration	Score 1 if sleep duration is 6 to 8 hours/day, otherwise 0. (Data-Field 1160)

Table S15. Incident obesity phenotype definition. Related to STAR Methods.

Category	Data-Field	Description	Coding
Reception	53	Date of attending assessment centre	
Hospital inpatient data	41270	Diagnoses - ICD10	E66
Hospital inpatient data	41280	Date of first in-patient diagnosis - ICD10	
Hospital inpatient data	41271	Diagnoses - ICD9	278
Hospital inpatient data	41281	Date of first in-patient diagnosis - ICD9	
Hospital inpatient data	41272	Operative procedures - OPCS4	G30.1-8, G28.4-5, G32.1, G33.1
Hospital inpatient data	41282	Date of first operative procedure - OPCS4	
Death Register records	40001	Underlying (primary) cause of death: ICD10	E66
Death Register records	40002	Contributory (secondary) causes of death: ICD10	E66
Death Register records	40000	Date of death	
First occurrences	130792	Date E66 first reported (obesity)	
First occurrences	130793	Source of report of E66 (obesity)	

Table S16. Definition of 20 obesity-related morbidities in UK Biobank. Related to STAR Methods.

Phenotype	ICD-9	ICD-10	OPCS-4	Self-reported data-fields (codes)
Cardiovascular diseases				
Coronary artery disease	410,411,412,4119,4129,4109	I21,I210,I211,I212,I213,I214,I219,I22,I220,I221,I228,I229,I23,I230,I231,I232,I233,I234,I235,I236,I238,I24,I240,I241,I248,I249,I252	K40,K401,K402,K403,K404,K408,K409,K41,K411,K412,K413,K414,K418,K419,K42,K421,K422,K423,K424,K428,K429,K43,K431,K432,K433,K434,K438,K439,K44,K441,K442,K448,K449,K451,K452,K453,K454,K455,K456,K458,K459,K46,K461,K462,K463,K464,K465,K468,K469,K471,K491,K492,K493,K494,K498,K499,K501,K502,K504,K751,K752,K753,K754,K758,K759	20002(1075), 20004(1095, 1070, 1523)
Hypertension	401.X	I10		20002(1065, 1072), 6150(4), 6177(2)
Heart failure	428.X	I11.0, I13.0, I13.2, I50.X		20002(1076)
Atrial fibrillation	427.3	I48		20002(1471)
Pulmonary embolism	415.1	I26.X		20002(1093), 6152(7)
Venous thromboembolism	451.1	I80.2	L90.2	20002(1068, 1093), 6152(5)
Aortic valve stenosis		I35.0, I35.2	K26.X	20002(1490)
Cancers				
Breast cancer (female only)	174.X	C50.X		20001(1002)
Endometrial cancer (female only)		C541		20001(1040)

Ovarian cancer (female only)	183.X	C56.X		20001(1039)
Colorectal cancer		C18.X, C19.X, C20.X		20001(1020, 1022, 1023)
Hepatocellular carcinoma		C22.0		20001(1024)
Gastrointestinal diseases				
Gastroesophageal reflux disease		K21.9, K21.0	G24.X, G25.X	20002(1138)
Cholelithiasis		K80.X		20002(1162)
Cholecystitis		K81.X		20002(1163)
Crohn's disease	555.X	K50.X		20002(1462)
Nonalcoholic fatty liver disease	571.8	K76.0		
Physically associated morbidities				
Stress urinary incontinence	625.6	N39.3		
Sleep apnea		G47.3		20002(1123)
Knee osteoarthritis	715.16, 715.36	M17.0, M17.1, M17.9	W40.X , W41.X, W42.X	

Table S17. List of weight-gaining medications. Related to STAR Methods.

**Category**	**UK Biobank data-field (codes)**
TCA antidepressants (amitriptyline (Elavil), doxepin (Silenor), nortriptyline (Pamelor))	20003 (1140879616, 1140867658, 1140867640, 1140867818)
Epilepsy medications (gabapentin (Gralise), pregabalin (Lyrica), and vigabatrin (Sabril))	20003 (1140872228, 1141200004, 1141200072, 2018602634, 1140872280, 1140872284)
Beta-blocker (atenolol (Tenormin) and metoprolol (Lopressor))	20003 (1140866738, 1140866756, 1140879818, 1140860274)
SSRI (escitalopram (Lexapro), paroxetine (Paxil), sertraline (Zoloft))	20003 (1141180212, 1140867888, 1140867878)
MAOI (phenelzine (Nardil))	20003 (1140867850, 1140910704, 1140867852)
Insulin	20003 (1140883066), 6177 (3), 6153 (3)
Sulfonylurea (gliclazide (Diamicron) and glibenclamide (Glynase))	20003 (1140874744, 1140874746, 1140874718)
Antipsychotics (olanzapine (Zyprexa))	20003 (1140928916)

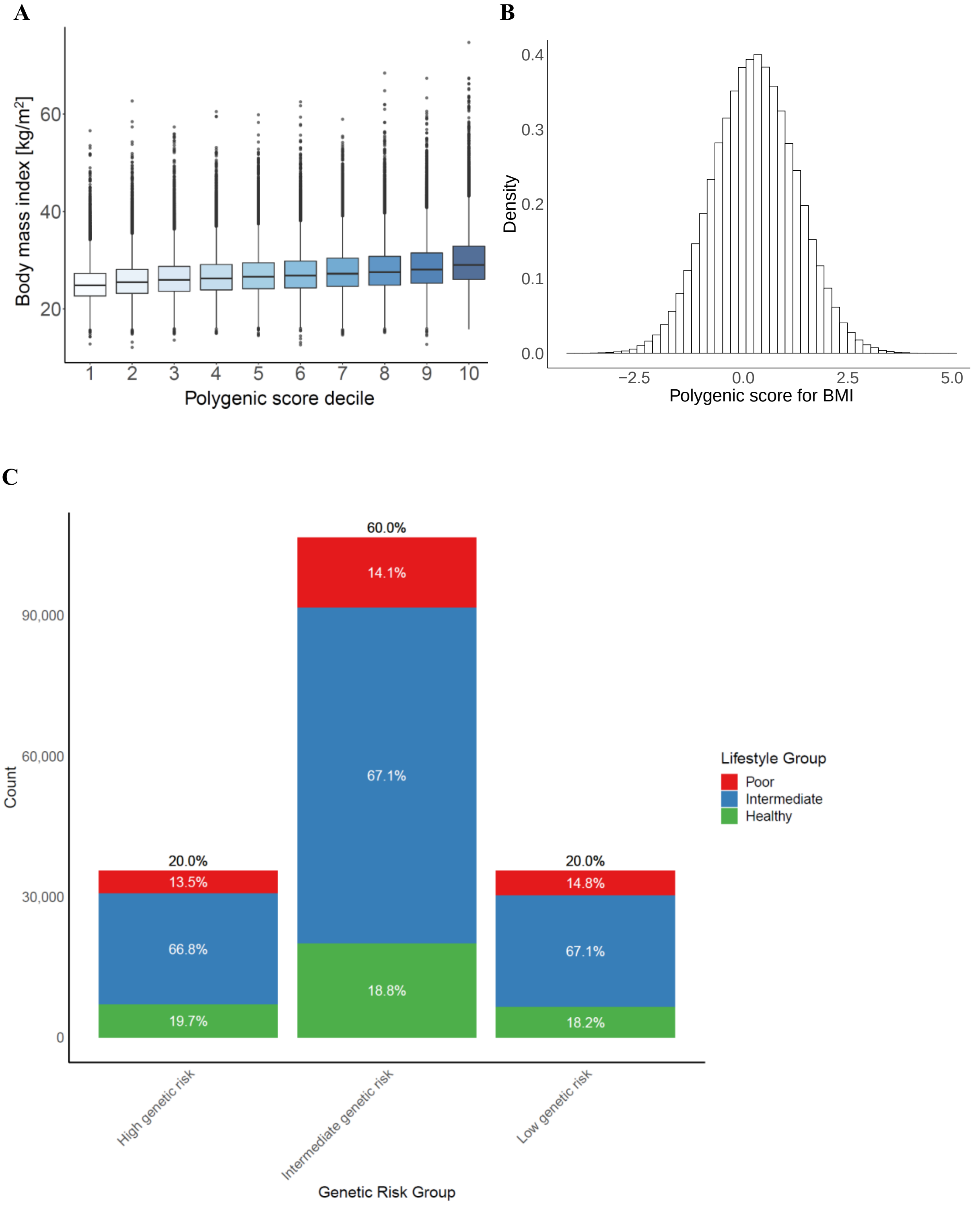

Figure S1. Polygenic scores for body mass index (BMI): association with measured BMI, distribution, and interaction with lifestyle risk factors. Related to STAR Methods. (A) Association between polygenic scores for BMI and measured BMI. The horizontal lines within each boxplot represent the median and the top/bottom of each box indicate the interquartile range. The whiskers reflect the maximum and minimum values within each group. (See also Data S1) (B) Distribution of polygenic scores (standardized) for BMI. (C) Distribution of lifestyle and genetic risk. Numbers in black above each bar refer to the percentage of individuals in each genetic risk category. For each genetic risk category, the percentage of individuals in each lifestyle risk category is displayed in white font.

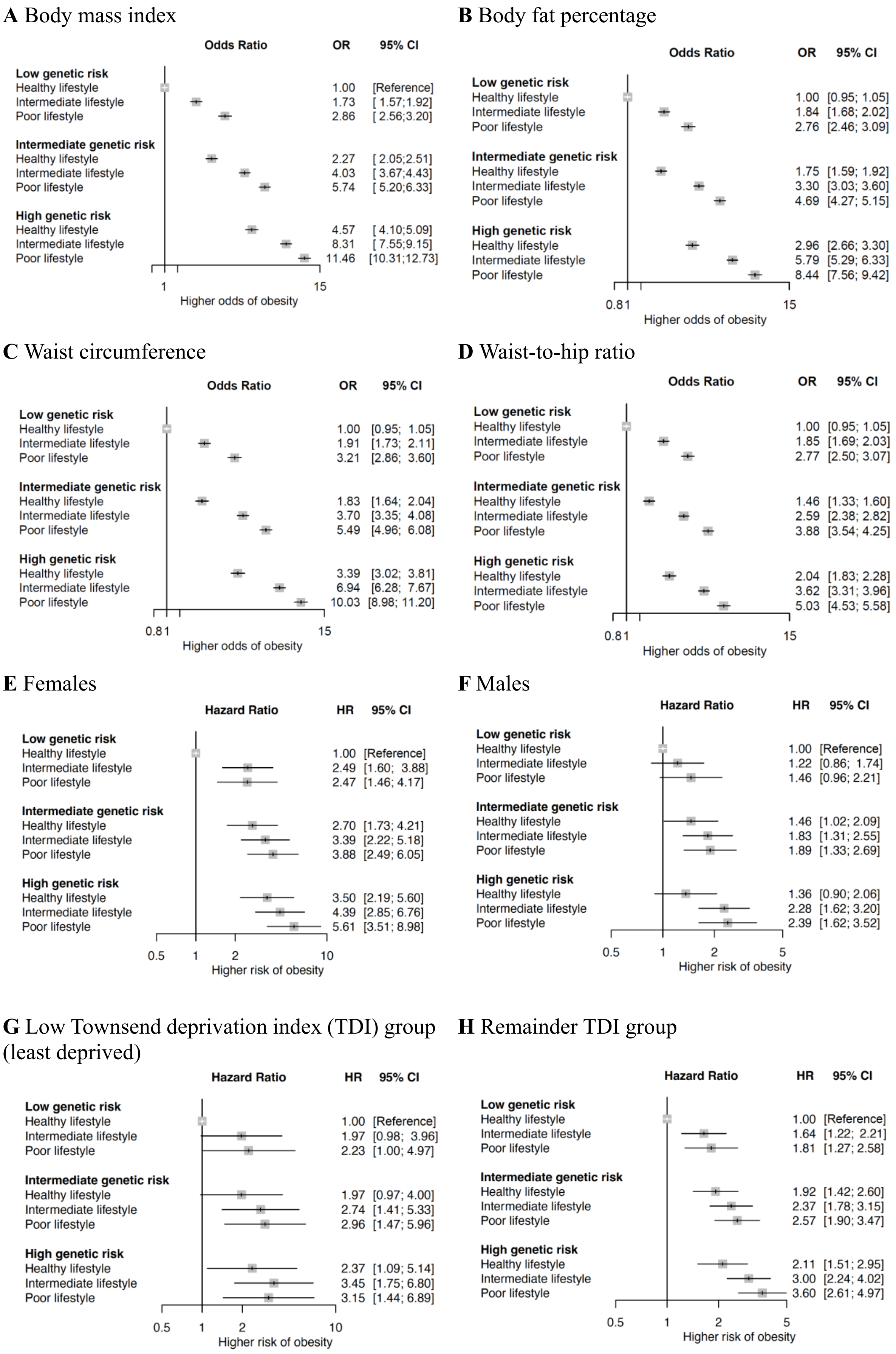
A Body mass index
Odds Ratio OR 95% CI
Low genetic risk
Healthy lifestyle 1.00 [Reference]
Intermediate lifestyle 1.73 [1.57;1.92]
Poor lifestyle 2.86 [2.56;3.20]
Intermediate genetic risk
Healthy lifestyle 2.27 [2.05;2.51]
Intermediate lifestyle 4.03 [3.67;4.43]
Poor lifestyle 5.74 [5.20;6.33]
High genetic risk
Healthy lifestyle 4.57 [4.10;5.09]
Intermediate lifestyle 8.31 [7.55;9.15]
Poor lifestyle 11.46 [10.31;12.73]
1
15
Higher odds of obesity
B Body fat percentage
Odds Ratio OR 95% CI
Low genetic risk
Healthy lifestyle 1.00 [0.95; 1.05]
Intermediate lifestyle 1.84 [1.68; 2.02]
Poor lifestyle 2.76 [2.46; 3.09]
Intermediate genetic risk
Healthy lifestyle 1.75 [1.59; 1.92]
Intermediate lifestyle 3.30 [3.03; 3.60]
Poor lifestyle 4.69 [4.27; 5.15]
High genetic risk
Healthy lifestyle 2.96 [2.66; 3.30]
Intermediate lifestyle 5.79 [5.29; 6.33]
Poor lifestyle 8.44 [7.56; 9.42]
0.81
15
Higher odds of obesity
C Waist circumference
Odds Ratio OR 95% CI
Low genetic risk
Healthy lifestyle 1.00 [0.95; 1.05]
Intermediate lifestyle 1.91 [1.73; 2.11]
Poor lifestyle 3.21 [2.86; 3.60]
Intermediate genetic risk
Healthy lifestyle 1.83 [1.64; 2.04]
Intermediate lifestyle 3.70 [3.35; 4.08]
Poor lifestyle 5.49 [4.96; 6.08]
High genetic risk
Healthy lifestyle 3.39 [3.02; 3.81]
Intermediate lifestyle 6.94 [6.28; 7.67]
Poor lifestyle 10.03 [8.98; 11.20]
0.81
15
Higher odds of obesity
D Waist-to-hip ratio
Odds Ratio OR 95% CI
Low genetic risk
Healthy lifestyle 1.00 [0.95; 1.05]
Intermediate lifestyle 1.85 [1.69; 2.03]
Poor lifestyle 2.77 [2.50; 3.07]
Intermediate genetic risk
Healthy lifestyle 1.46 [1.33; 1.60]
Intermediate lifestyle 2.59 [2.38; 2.82]
Poor lifestyle 3.88 [3.54; 4.25]
High genetic risk
Healthy lifestyle 2.04 [1.83; 2.28]
Intermediate lifestyle 3.62 [3.31; 3.96]
Poor lifestyle 5.03 [4.53; 5.58]
0.81
15
Higher odds of obesity
E Females
Hazard Ratio HR 95% CI
Low genetic risk
Healthy lifestyle 1.00 [Reference]
Intermediate lifestyle 2.49 [1.60; 3.88]
Poor lifestyle 2.47 [1.46; 4.17]
Intermediate genetic risk
Healthy lifestyle 2.70 [1.73; 4.21]
Intermediate lifestyle 3.39 [2.22; 5.18]
Poor lifestyle 3.88 [2.49; 6.05]
High genetic risk
Healthy lifestyle 3.50 [2.19; 5.60]
Intermediate lifestyle 4.39 [2.85; 6.76]
Poor lifestyle 5.61 [3.51; 8.98]
0.5 1 2 10
Higher risk of obesity
F Males
Hazard Ratio HR 95% CI
Low genetic risk
Healthy lifestyle 1.00 [Reference]
Intermediate lifestyle 1.22 [0.86; 1.74]
Poor lifestyle 1.46 [0.96; 2.21]
Intermediate genetic risk
Healthy lifestyle 1.46 [1.02; 2.09]
Intermediate lifestyle 1.83 [1.31; 2.55]
Poor lifestyle 1.89 [1.33; 2.69]
High genetic risk
Healthy lifestyle 1.36 [0.90; 2.06]
Intermediate lifestyle 2.28 [1.62; 3.20]
Poor lifestyle 2.39 [1.62; 3.52]
0.5 1 2 5
Higher risk of obesity
G Low Townsend deprivation index (TDI) group (least deprived)
Hazard Ratio HR 95% CI
Low genetic risk
Healthy lifestyle 1.00 [Reference]
Intermediate lifestyle 1.97 [0.98; 3.96]
Poor lifestyle 2.23 [1.00; 4.97]
Intermediate genetic risk
Healthy lifestyle 1.97 [0.97; 4.00]
Intermediate lifestyle 2.74 [1.41; 5.33]
Poor lifestyle 2.96 [1.47; 5.96]
High genetic risk
Healthy lifestyle 2.37 [1.09; 5.14]
Intermediate lifestyle 3.45 [1.75; 6.80]
Poor lifestyle 3.15 [1.44; 6.89]
0.5 1 2 10
Higher risk of obesity
H Remainder TDI group
Hazard Ratio HR 95% CI
Low genetic risk
Healthy lifestyle 1.00 [Reference]
Intermediate lifestyle 1.64 [1.22; 2.21]
Poor lifestyle 1.81 [1.27; 2.58]
Intermediate genetic risk
Healthy lifestyle 1.92 [1.42; 2.60]
Intermediate lifestyle 2.37 [1.78; 3.15]
Poor lifestyle 2.57 [1.90; 3.47]
High genetic risk
Healthy lifestyle 2.11 [1.51; 2.95]
Intermediate lifestyle 3.00 [2.24; 4.02]
Poor lifestyle 3.60 [2.61; 4.97]
0.5 1 2 5
Higher risk of obesity

Figure S2. Association of genetic risk and lifestyle with obesity across different subgroups. Related to Figure 2. Association of genetic risk and lifestyle with obesity measured by (A) body mass index, (B) body fat percentage, (C) waist circumference, and (D) waist-to-hip ratio at baseline. Given that there is no time-to-event variable for aforementioned obesity measures, we used linear regression; adjusted odds ratios (OR) for the risk of obesity according to genetic and lifestyle risks are shown. Association of genetic risk and lifestyle with incident obesity in (E) females, (F) males, (G) low Townsend deprivation index (TDI) group (least deprived) and (H) remainder TDI group. Adjusted hazard ratios (HR) for the risk of incident obesity according to genetic risk and lifestyle risk in each sex and different socioeconomic status are shown. Participants with low genetic risk and a healthy lifestyle served as the reference group. CI, confidence interval.

A

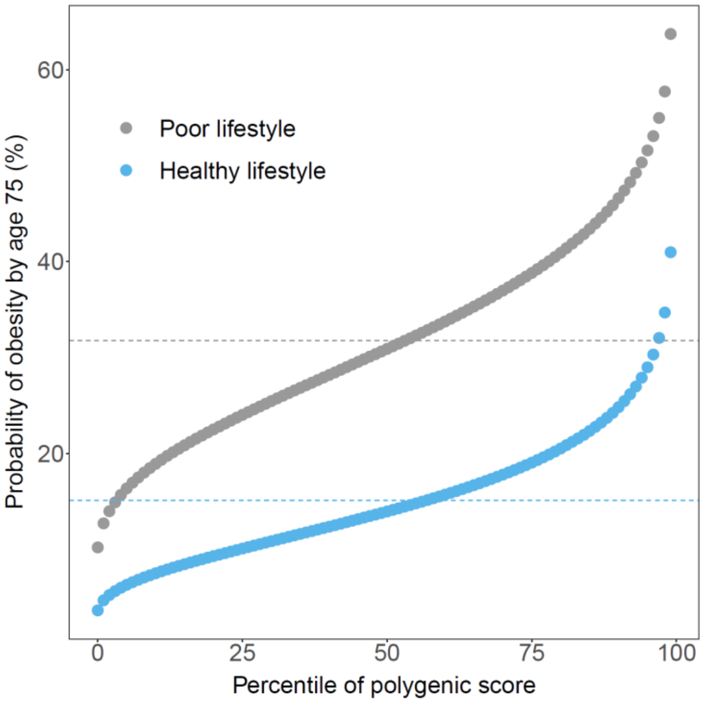

B

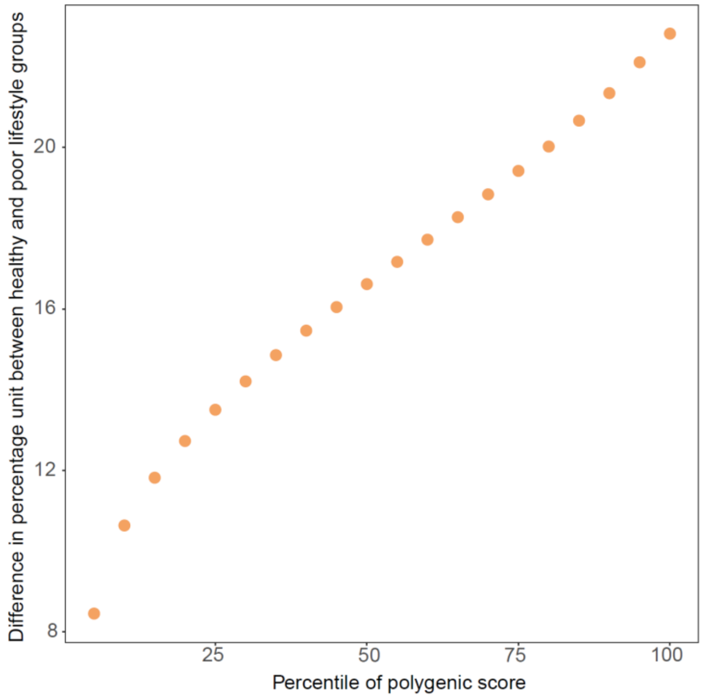

Figure S3. Predicted probability of obesity by age 75 years based on prevalent obesity (BMI ≥ 30). Related to Figure 3. (A) Probability of obesity risk (%) by polygenic score for healthy (blue) and poor (black) lifestyle groups. (B) The difference in percentage unit between healthy and poor lifestyle groups by polygenic score. See also Data S1. Related to Figure 3.

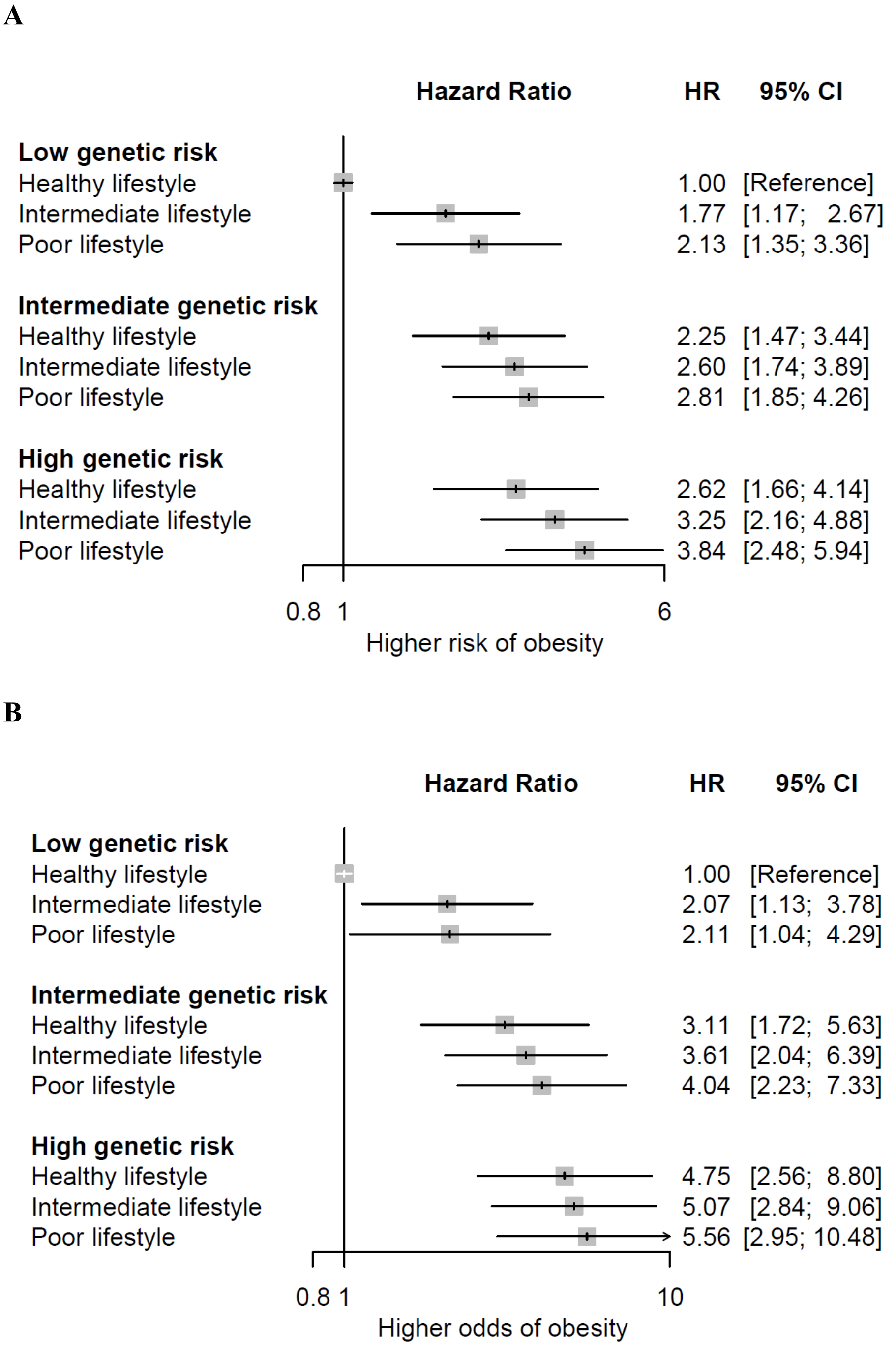

Figure S4. Associations of genetic risk and weighted lifestyle scores with incident obesity based on diagnosis codes and repeated BMI measurements. Related to Figure 2. (A) Association of genetic risk and weighted lifestyle scores with incident obesity (diagnosis code). We constructed a weighted lifestyle score based on the five lifestyle factors by using the equation: weighted lifestyle score = (b1*factor1 + b2*factor 2 +. . .+ b5*factor 5) * (5/sum of the b coefficients). This weighted score also ranges from 0 to 5 points. Effect estimates were derived from adjusted models. (B) Association of genetic risk and lifestyle scores with incident obesity (based on repeated BMI measures). Among 21,715 participants with follow-up BMI measurements and complete lifestyle factor data, 1,202 individuals transitioned to a BMI ≥ 30 during the follow-up period. Effect estimates were derived from adjusted models. Participants with low genetic risk and a healthy lifestyle served as the reference group. CI, confidence interval.
